# Adaptation strategies for nurses with musculoskeletal pain in hospital work: a systematic review

**DOI:** 10.1590/1518-8345.7812.4725

**Published:** 2025-11-10

**Authors:** Jorge Gabriel Tuz-Colli, Yolanda Flores-Peña, Heloisa Ehmke Cardoso dos Santos, Fernanda Ludmilla Rossi Rocha, Maria Helena Palucci Marziale

**Affiliations:** 1 Universidad Autónoma de Nuevo León, Facultad de Enfermería de Nuevo León, Monterrey, Nuevo León, México. Universidad Autónoma de Nuevo León Facultad de Enfermería de Nuevo León Nuevo León Monterrey México; 2 Universidade de São Paulo, Escola de Enfermagem de Ribeirão Preto, Collaborating Centre for Nursing Research Development, Ribeirão Preto, SP, Brazil. Universidade de São Paulo Escola de Enfermagem de Ribeirão Preto Collaborating Centre for Nursing Research Development SP Ribeirão Preto Brazil

**Keywords:** Musculoskeletal Pain, Coping Skills, Working Conditions, Ergonomics, Nurse Practitioners, Nursing

## Abstract

to analyze the effectiveness of individual coping strategies and hospital work environment adaptations for nurses with musculoskeletal pain.

a systematic review was conducted following the Preferred Reporting Items for Systematic reviews and Meta-Analyses (PRISMA) guidelines. The search was performed in six databases, including experimental and pre-experimental studies. Risk of bias was assessed using the RoB2 (Risk of Bias 2) and ROBINS-I (Risk of Bias In Non-randomized Studies of Interventions) tools. Methodological quality was evaluated using the Joanna Briggs Institute model, the Jadad Scale and the Melnyk, Buck and Gallagher-Ford Levels of Evidence.

eight studies were included, identifying individual coping strategies such as stretching exercises, auriculotherapy and mindfulness. Workplace-based strategies were multidisciplinary, integrated and focused on training for identifying and preventing musculoskeletal risks while improving working conditions.

the most effective strategies combine workers’ individual actions with improvements in working conditions, addressing physical, psychosocial and ergonomic factors to adapt the work environment, prevent musculoskeletal pain progression and maintain job performance. PROSPERO registration number: CRD42024575014.

## Abstract (key points)

**(1)** Individual and workplace strategies have been implemented to reduce musculoskeletal pain (MSP). **(2)** Non-pharmacological strategies such as mindfulness and auriculotherapy reduce MSP. **(3)** Applying ergonomics in the workplace reduces MSP. **(4)** Workers’ individual needs must be considered to reduce MSP.

## Introduction

Musculoskeletal pain (MSP) affects bones, muscles, ligaments, tendons and nerves, representing the primary symptom of musculoskeletal disorders (MSDs). It is more usual in the back, neck and shoulder regions and is associated with conditions such as osteoarthritis and rheumatoid arthritis, as well as with pain caused by muscle strains, fractures, trauma and other injuries^(1-2)^.

MSDs accompanied by MSP are a major Public Health issue affecting the working population, particularly health professionals, with Nursing ones showing the highest prevalence. These disorders are the leading cause of work absenteeism, functional limitations and permanent disability among Nursing staff. Additionally, they oftentimes restrict mobility and dexterity, reducing work capacity, increasing absenteeism and early retirements, raising healthcare costs, compromising patient safety and reducing quality of life^(3-5)^.

The term “Nursing professionals” refers to qualified and licensed individuals practicing Nursing (including nurses, technicians, assistants and support staff), as defined by each country’s regulations. This category represents approximately 59% of all healthcare workers and constitutes the largest workforce in hospitals^(6)^. Due to their high representation, diverse and demanding responsibilities and constant exposure to occupational hazards—such as patient handling, heavy lifting and participation in clinical procedures—hospital nurses are particularly vulnerable to developing MSDs^(7)^.

A multicenter study in emerging economies^(8)^ revealed an MSD prevalence of 37% among the general workforce but rising to 92% among hospital nurses. Meta-analyses^(9)^ indicate high prevalence rates: 64% for lower back pain and 40% for neck and shoulder pain^(10)^. Furthermore, 33% of all sick leaves in this group are linked to MSDs^(10)^. Low back pain stands out as the primary contributor to the overall burden of these disorders and is the most common cause of disability in 160 countries^(3,11)^.

Preventing work-related MSDs is crucial and should be addressed through preventive workplace measures for Nursing professionals alongside adaptation strategies enabling affected workers with MSP to remain active. Pain management is essential for quality of life and daily functioning; however, research has predominantly focused on MSP prevalence, risk factors (psychosocial, demographic, physical and mental) or on prevention interventions whose real-world implementation remains suboptimal^(12)^—despite MSP’s persistently high global prevalence even in high-income economies^(13-15)^.

The few studies on coping strategies for MSP among Nursing professionals indicate the use of various strategies, some of them even not recommended. Among the most commonly used strategies is resorting to analgesics such as Acetaminophen and non-steroidal anti-inflammatory drugs (NSAIDs). Other strategies include ignoring pain, seeking medical consultations with specialists, resorting to alternative medications, complementary therapies such as therapeutic massages, aromatherapy, acupuncture, chiropractics and *reiki*; self-managing pain, bed rest; physiotherapy and exercises for pain management^(8,16-17)^.

Recent research studies have highlighted the relationship of individual/psychosocial factors and ergonomics with the development of work-related musculoskeletal disorders. This finding contrasts with the traditional view that attributed these injuries primarily to exposure to biomechanical factors^(18-20)^.

Ergonomics seeks to adapt work environments to the workers’ needs^(21-22)^. In the hospital setting, applying ergonomic principles can prevent onset and progression of MSDs and MSP, as well as improve Nursing professionals’ safety and health and, consequently, their work-related quality of life^(19,20,23)^.

Coping with MSP is essential to preserving productivity among Nursing professionals, whose performance not only represents their economic livelihood but also a necessary workforce to meet the growing demand for health services. This is exacerbated by the global shortage of 5.9 million Nursing professionals, particularly in regions such as Africa, Southeast Asia and the Eastern Mediterranean, as well as in Latin American countries like the Dominican Republic, Honduras, Guyana and Venezuela, as reported in 2020 by the Pan American Health Organization (PAHO)^(6)^.

To alleviate MSP, Nursing professionals employ both individual and workplace coping strategies to maintain their performance and prevent progression of the problem. However, full recovery and safe reintegration into their work activities require employers and managers to implement strategies such as providing patient transfer equipment and introducing flexible schedules and adapting working conditions to the workers’ needs and capabilities (applying ergonomics). These adaptations reduce the risk of exacerbations and accidents, especially in workers with reduced functional capacity^(24)^.

However, according to the literature review, it was found that the available information regarding individual coping strategies, as well as workplace adaptation strategies for Nursing professionals, is limited. Therefore, this systematic review was conducted with the objective of analyzing the effectiveness of individual coping strategies and adaptations to the hospital work environment for Nursing professionals experiencing musculoskeletal pain.

## Method

The Systematic Literature Review protocol was registered on the International Prospective Register of Systematic Reviews (PROSPERO), Registration Number CRD42024575014 dated August 16th, 2024. This review was conducted in accordance with the guidelines provided by the Centre for Reviews and Dissemination (CRD)^(25)^ and the Preferred Reporting Items for Systematic Reviews and Meta-Analyses (PRISMA)^(26)^. The review followed these stages: a) Definition of the research question, b) Review protocol: inclusion and exclusion criteria, c) Literature search: development of the search strategy, d) Selection of studies, e) Data extraction, f) Critical assessment regarding the quality of the studies included, g) Synthesis of the results and h) Presentation and dissemination of the results.

### Definition of the research question

The research question was as follows: “How effective are individual coping strategies and adaptations in the hospital work environment for Nursing professionals experiencing musculoskeletal pain?”. The question was structured using the PICOS framework^(27)^ (Population, Interventions, Comparison, Outcome, Study design), where P (Population): Nursing professionals working in hospitals (nurses, nursing technicians and nursing assistants) with MSP; I (Intervention): Individual coping strategies and workplace adaptations for Nursing professionals with MSP; C (Comparison/Control): Not applicable; O (Outcome): Nursing professionals’ adaptation in the workplace.

Definitions: a) Nursing professionals: Nurses, nursing technicians and nursing assistants. b) MSP: An unpleasant sensory and emotional experience affecting bones, muscles, ligaments, tendons and nerves, representing the primary symptom in musculoskeletal disorders.

### Review protocol: inclusion and exclusion criteria

Inclusion criteria: Primary studies addressing the research question about the effectiveness of individual coping strategies and workplace adaptations for Nursing professionals with MSP. Studies with experimental designs (randomized controlled trials - RCTs, clinical trials - CTs) and pre-experimental designs (pre- and post-test with a single group) were included. The articles were not restricted by language and were published between January 2000 and July 2024.

Exclusion criteria: Studies not addressing the research question, Nursing professionals not working in hospitals, pilot studies, descriptive studies, observational studies and all types of literature reviews.

### Literature search: development of the search strategy

The searches were conducted in the following databases: MEDLINE/PubMed^®^, Web of Science, Scopus, Cochrane Library, Cumulative Index to Nursing and Allied Health Literature (CINAHL) and Latin American and Caribbean Health Sciences Literature (*Literatura Latinoamericana y del Caribe en Ciencias de la Salud* - LILACS). The search strategy combined controlled descriptors and keywords using Boolean operators “OR” for synonyms and similar terms and “AND” for keywords, tailored to the specificities of each database. A professional librarian provided technical guidance in developing the search strategy, which was structured using controlled vocabularies from the Medical Subject Headings (MeSH) and the Descriptors in Health Sciences (*Descriptores en Ciencias de la Salud*, DeCS). The searches were performed between August 31^st^ and September 7^th^, 2024, and included articles published between January 2000 and July 2024. The references were managed in the Mendeley software. Figure 1 illustrates the search strategy.

**Figure 1-:** Search strategies. Ribeirão Preto, SP, Brazil, 2024

PubMed/MEDLINE
(“Nursing Assistant*” OR “Personnel nursing” OR Nurse* OR “nursing worker*” OR “nursing professional*”) AND (“Musculoskeletal Pain”[Mesh] OR “Musculoskeletal Pain” OR “muscle pain”)
Web of Science
(“Nursing Assistant*” OR “Personnel nursing” OR Nurse* OR “nursing worker*” OR “nursing professional*”) AND (“Musculoskeletal Pain” OR “muscle pain”)
Scopus
(“Nursing Assistant*” OR “Personnel nursing” OR Nurse* OR “nursing worker*” OR “nursing professional*”) AND (“Musculoskeletal Pain” OR “muscle pain”)
Cochrane
((MeSH descriptor: [Nursing NEXT Assistant*] explode all trees) OR (Personnel NEXT nursing) OR Nurse OR Nurses OR (nursing NEXT worker*) OR (nursing NEXT professional*)) AND ((MeSH descriptor: [Musculoskeletal Pain] explode all trees) OR “Musculoskeletal Pain” OR “muscle pain”)
CINAHL
(“Nursing Assistant*” OR “Personnel nursing” OR Nurse* OR “nursing worker*” OR “nursing professional*”) AND ((MH “Musculoskeletal Pain”) OR “Musculoskeletal Pain” OR “muscle pain”)
BVS – LILACS
(“Profissional de enfermagem” OR “Profissionais de enfermagem” OR “Profissionais da enfermagem” OR “Técnico de enfermagem” OR “trabalhadores da enfermagem” OR “trabalhador da enfermagem” OR “trabalhador de enfermagem” OR “trabajadores de enfermería” OR “trabajador de enfermería” OR “Assistentes de Enfermagem” OR “Auxiliar de Enfermagem” OR “Auxiliar do Enfermeiro” OR “Auxiliares de Enfermagem” OR “Auxiliares de Enfermeiros” OR “Asistentes de Enfermería” OR “asistente de enfermería” OR “auxiliar de enfermería” OR “auxiliar del enfermero” OR “auxiliares de enfermeros” OR “auxiliares de enfermería” OR enfermeri* OR Enfermero* OR Enfermera* OR “Healthcare Assistant*” OR “Nursing Assistant*” OR “Personnel nursing” OR “nursing professional* OR Nurse* OR”nursing worker*” OR “nursing professional*” OR Enfermagem OR Enfermería OR Nursing) AND (“Dor Musculoesquelética*” OR “Dores Musculoesqueléticas*” OR “Dor muscular*” OR “Dores musculares” OR “Dor Osteomuscular*” OR “dolor osteomuscular*” OR “Dolor Musculoesquelético” OR “Musculoskeletal Pain” OR “muscle pain”)

### Selection of studies

The search results from the databases were exported to the Rayyan^®^ online tool^(28)^, which was used to store, organize, remove duplicates and blindly select the studies. The study selection process was conducted in two phases. In the first one, titles and abstracts were analyzed independently and anonymously by two reviewers, following the steps outlined in the PRISMA flowchart^(26)^. In the second phase, the full texts of the articles selected were analyzed and the inclusion criteria were applied in a masked manner (reviewers R1 and R2) to minimize bias in the review process (September 2024). Discrepancies regarding study selection were solved by a third reviewer (R3) by discussing and reviewing the selection criteria.

a) The reviewers employed the following definitions to guide their decisions:

b) Individual coping strategies for workers with MSP: they are dynamic cognitive and behavioral actions that Nursing professionals develop to address stressful situations, alleviate MSP and maintain their workplace activities^(29)^.

Workplace adaptation for workers with MSP: achieved through workplace interventions (e.g.: activities, tasks and programs) designed to modify or reduce occupational risks and enhance workers’ knowledge, behaviors and attitudes. These interventions may be implemented directly in the workplace or targeted at workers through education or continuous training and adapting the workplace to Nursing professionals’ psychophysiological characteristics^(30)^.

### Data extraction

A data extraction table was developed in *Microsoft Excel*^®^ to extract information from the studies selected. This table included the following details: publication year, country of origin, study objective, study design type, inclusion and exclusion criteria for the sample, data analysis type and method, description of the intervention, assessment tools, results, conclusions and level of evidence.

The most relevant findings and conclusions of the studies selected were analyzed in relation to the review question, categorized into two predefined topics:

a) Interventions and/or coping strategies implemented by Nursing professionals with work-related MSP.

b) Interventions and/or strategies to promote the adaptation of Nursing professionals with MSP implemented in the workplace.

### Critical assessment regarding the quality of the studies included

A number of tools were used to evaluate the methodological quality of the studies under review, considering both their methodology and content. For this purpose, the critical appraisal tools from the Joanna Briggs Institute (JBI)^(31)^ were applied. The Jadad scale^(32)^ was used to assess the methodological quality of randomized controlled trials (RCTs). This scale evaluates three study items through five binary questions with “Yes” (worth 1 point) or “No” (worth 0 points) answers. The items assess randomization, blinding and participant dropouts or withdrawals, with a total score from 0 to 5. Scores from 0 to 2 are considered “low methodological quality”, while scores between 3 and 5 are deemed as with “high methodological quality”.

To determine the level of evidence corresponding to the studies selected, the framework established by Melnyk, Buck and Gallagher-Ford^(33)^ was followed. This step was also conducted independently and in a masked manner by the reviewers. A third reviewer was called upon to solve any conflicts in the evaluation.

### Risk of bias assessment

The risk of bias in the randomized clinical trials was assessed independently by two reviewers using the Risk of Bias 2 (RoB2) tool^(34)^. For the non-randomized pre-experimental clinical studies, the Risk of Bias in Non-Randomized Studies of Interventions (ROBINS-I) tool^(35)^ was employed.

### Ethical aspects

This secondary study did not require approval from any Research Ethics Committee. Nonetheless, the authors declare no conflicts of interest that could compromise the analysis of the results obtained in this paper.

## Results

A total of 1,732 studies were retrieved: 316 (18.2%) articles from PubMed/Medline, 435 (25.1%) from Scopus, 417 (24.1%) from Web of Science, 281 (16.2%) from Cochrane Library, 213 (12.3%) from CINAHL and 70 (4%) from LILACS. After removing duplicate records (n=711), 1,021 studies were identified for title and abstract screening. This process left 33 publications as potential candidates for full-text review. Following the full-text assessment, eight scientific articles were ultimately included in this study.

**Figure 2-:**
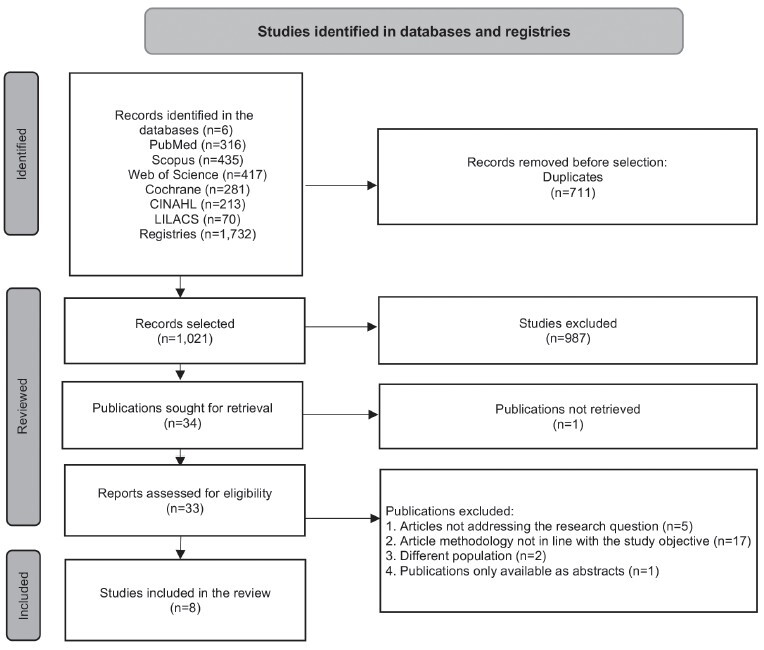
PRISMA flowchart showing the literature review process^(26)^

Figure 2 presents the PRISMA flowchart, showing the literature review process^(26)^.

Regarding the characteristics of the studies, all articles were published in English. Of the studies included, 3 (37.5%) were conducted in Brazil, 2 (25.0%) in Japan, 1 (12.5%) in Spain, 1 (12.5%) in Finland and 1 (12.5%) in Turkey. Concerning the study populations, in 4 (50.0%) studies, the participants were exclusively nurses, while in the remaining 4 (50.0%), the participants were healthcare workers, primarily including nurses.

### Synthesis of the evidence

The analysis corresponding to the level of evidence of the studies was conducted using the framework established by Melnyk, Buck and Gallagher-Ford. All eight studies were classified as Level II evidence, including seven randomized controlled trials (RCTs)^(36-42)^ and one pre-experimental study^(43)^.

In the methodological quality assessment of the seven RCTs using the Jadad scale^(32)^, six (85.7%) studies were found to be of high methodological quality^(36-39,41-42)^, while one (14.3%) was rated as low quality^(40)^. The pre-experimental study^(43)^ was classified as having low methodological quality.

The instruments used to assess MSP varied across the studies and included the Visual Analog Scale (VAS), adaptations of the Nordic Musculoskeletal Questionnaire and the Fear-Avoidance Beliefs Questionnaire (FABQ).

The studies analyzed evaluated the effectiveness of various interventions implemented in hospital settings, targeting Nursing professionals with work-related MSP. The reported individual coping strategies included therapeutic exercises, Pilates, active and progressive stretching^(36-39)^; therapies such as adapted mindfulness^(40)^; auriculotherapy^(41)^; and pain neuroscience education^(39)^. The interventions aimed at promoting workplace adaptation included a multifaceted intervention with a participatory approach across the three prevention levels^(42)^ and an ergonomic risk management program^(43)^.

**Figure 3-:**
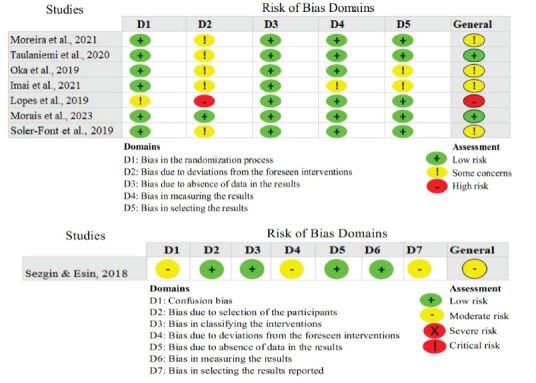
Risk of bias assessment of the studies included using the RoB2 (Risk of Bias 2) and ROBINS-I (Risk of Bias in Non-Randomized Studies of Interventions) tools. Ribeirão Preto, SP, Brazil, 2024

Figure 3 presents the results of the risk-of-bias assessment for the studies included.

Figures 4 and 5 present the characteristics of the studies included in the review.

**Figure 4–:** Characteristics of the studies included in the review on coping strategies or actions implemented by Nursing staff for work-related musculoskeletal disorders (n = 8). Ribeirão Preto, SP, Brazil, 2024

First author, year, country	Study design	Level of evidence/ Methodological quality	Participants	Intervention	Outcomes
Moreira, et al., 2021 ^( 36 )^ ; Brazil	Experimental, randomized controlled clinical trial, single-blind, parallel-group with allocation concealment	Level II 3	IG*: 46 nursing assistants; and 44 from the CG ^†^	The program was applied twice a week for 30 minutes per session. The training consisted of exercises for spinal stabilization. Each session was divided into three steps: 1) Five minutes of warm-up exercises; 2) 20 minutes of therapeutic exercises; 3) Five minutes of hamstring stretching and cool-down exercises at the end of the session.	The participants showed improvements in trunk flexor muscle strength with an effect size of 0.77 ( *p* =0.002) and in the control of lower back symptoms by 6.25 ( *p* =0.002) after the therapeutic exercise program. The positive association between exercise exposure and symptom improvement was 2.04 times higher for the IG* than for the CG ^†^ (OR ^‡^ =2.04; 95% CI ^§^ 1.82-2.72).
Taulaniemi, et al., 2020 ^( 37 )^ ; Finland	Experimental, four-arm randomized controlled clinical trial	Level II 3	IG*: 165 healthcare workers and 54 in the CG ^†^ . Of these, 87% were nurses	A Pilates-based exercise program focused on controlling the neutral posture of the spine, divided into three stages. Stage 1. Supervised neuromuscular exercise classes. Stages 2 and 3: One supervised class and one home session with the aid of a DVD or a study-specific booklet. During this stage, the participants were allowed to exercise in supervised group sessions.	Practicing Pilates with good adherence reduced work-related fear-avoidance beliefs (FABs) and decreased low back pain intensity ( *r* =0.16, *p* =0.05). The study identified that motivational strategies should target individuals with low schooling levels, lower physical fitness and high FAB levels to achieve better exercise adherence.
Oka, et al., 2019 ^( 38 )^ ; Japan	Analytical, prospective, randomized, parallel-group, multicenter study with centralized evaluations	Level II 3	4,767 nurses 1799 in group A, 1420 in group B, and 1548 in Group C from 12 hospitals	Group A served as the Control Group. The participants in groups B and C received an exercise manual detailing how to perform the standing back extension exercise “One Stretch,” along with a 30-minute seminar. In Group B, participants only attended the 30-minute seminar. In Group C, both physical and psychological approaches were incorporated into the treatment of low back pain.	The standing back extension exercise called “One Stretch” effectively improved and prevented low back pain. The improvement rates were 13.3%, 23.5% and 22.6% in groups A, B and C, respectively. The pain worsening rates were 13.0%, 9.6% and 8.1%, respectively, decreasing as the intervention level increased (p<0.001).
Imai, et al., 2021 ^( 39 )^ ; Japan	Experimental, randomized controlled parallel-group trial	Level II 3	104 participants 53 in the IG* and 51 in the CG ^†^ , including healthcare workers such as nurses	The program consisted of a six-month plan involving progressive exercises and pain neuroscience education (PNE). The stretching sessions lasted 20 minutes and the walking sessions lasted approximately 30 minutes, performed 3-4 times per week. The Control Group received general feedback after completing a questionnaire.	The study demonstrated that a combination of PNE and exercise yielded better outcomes for patients, including reduced presenteeism ( *F* =12.87, *p* <0.001, η²=0.94), lower pain intensity ( *F* =11.0, *p* <0.001, η²=0.1), decreased physical stress and improved psychological status and quality of life.
Lopes, et al., 2019 ^( 40 )^ ; Brazil	Experimental, prospective, open-label trial with repeated measures	Level II 2	IG*: 64 nursing technicians No CG ^†^ was included	An adapted mindfulness program, consisting of weekly 60-minute sessions over 8 consecutive weeks. The participants were guided to practice daily meditation at home for 20 minutes, incorporating mindfulness into their daily routines and activities as an informal practice. During face-to-face meetings, exercises focused on pain management, breathing techniques, body scan, mindful walking, mindful movements with light body postures, sitting, lying down and meditation.	The mindfulness program demonstrated significant reductions in musculoskeletal symptoms, anxiety, depression and catastrophic pain ( *p* <0.001). A particularly significant finding was the reduction in catastrophic pain levels (η²=0.203; *p* <0.001) and MSP (η²=0.200; *p* <0.001). The positive effects were observed after 8 weeks and remained stable through the 12-week follow-up period.
Morais, et al., 2023 ^( 41 )^ ; Brazil	Experimental, randomized clinical trial, triple-blind (patient, statistician and outcome evaluators)	Level II 5	IG*: 34 workers; CG ^†^ : 33 workers, including healthcare personnel (e.g.: Nursing staff) diagnosed with chronic spinal pain	The participants received eight sessions of seed-based auriculotherapy, administered twice a week and lasting a mean of 10 to 15 minutes per session. The workers were instructed to leave the seeds in place for three days, manually stimulating them at least three times a day, applying pressure 15 times on each auricular point.	Seed-based auriculotherapy demonstrated positive therapeutic effects in reducing pain intensity among healthcare workers with chronic spinal pain. The IG* showed a significant 34% reduction in pain when compared to the CG ^†^ ( *p* =0.007). Regarding pain interference in daily living activities, a reduction in mean values was observed in both groups ( *p* <0.05). Additionally, a significant reduction in medication use was noticed ( *p* =0.013).
Soler-Font, et al., 2019 ^( 42 )^ ; Spain	Experimental, cluster-randomized controlled trial with two arms and a control group	Level II 3	IG*: 138 nurses CG ^†^ : 119 nurses	The intervention incorporated components targeting all three prevention levels. The primary prevention included: 1) Occupational risk factors to protect healthy workers from MSDs and MSD-related absenteeism. through participatory ergonomics; and 2) Promotion of healthy lifestyles at work (physical activity, emotional well-being and diet). Secondary and tertiary prevention involved a case management service for the early identification of MSPs.	The intervention was effective in reducing pain by up to 63% (OR ^‡^ =0.37; 95% CI ^§^ : 0.14-0.96) in the neck, shoulders and upper back among Nursing staff. A non-statistically significant reduction in lower back pain was observed in the Intervention Group when compared to the Control Group. Work functioning remained stable over the 12-month follow-up period in the Intervention Group, whereas a decline was noticed in the Control Group ( *p* <0.05).
Sezgin & Esin, 2018 ^( 43 )^ ; Turkey	Pre-experimental study with pretest and posttest for non-equivalent control groups	Level II 2	61 nurses, 30 in the IG* and 31 in the CG ^†^	The Ergonomic Risk Management Program (ERMP) consisted of two components: 1) A two-week video-based training focused on musculoskeletal risks for ICU nurses, complemented with preventive exercises; and 2) Personal interviews with nurses aimed at identifying predisposing, reinforcing and facilitating factors for behavioral change.	ERMP was effective in increasing exercise frequency ( *p* =0.017) and reducing musculoskeletal pain ( *p* =0.017) as well as ergonomic risk levels among ICU nurses ( *p* <0.05).

*IG = Intervention Group; ^†^CG = Control Group; ^‡^OR = Odds Ratio; ^§^CI = Confidence Interval

**Figure 5-:** Description of the interventions applied in the studies included (n = 8). Ribeirão Preto, SP, Brazil, 2024

First author, year, country	Intervention/technique used	Number of sessions	Duration per session	Evaluation instrument/technique	Outcome
Moreira, et al., 2021 ^( 36 )^ ; Brazil	Therapeutic exercise for spine muscle strength (warm-up, strengthening and stretching)	Mean of 17.5 sessions	30 minutes	Dynamometry: Strength of trunk flexor muscles; pressure pain threshold of the long dorsal muscle	Increased strength of flexor muscles Significant reduction in pressure pain threshold
Taulaniemi, et al., 2020 ^( 37 )^ ; Finland	Pilates-type physical exercise Neuromuscular exercise	Twice a week for the first two months, then once a week for four follow-up months	60 minutes	-Fear-Avoidance Beliefs Questionnaire (FABQ) -Exercise adherence log	Reduced beliefs related to avoiding pain associated with physical activity and work Greater adherence to exercise
Oka, et al., 2019 ^( 38 )^ ; Japan	Active spinal extension exercises called “One Stretch” Educational seminar Psychological and physical approaches	1 seminar and 6-month follow-up	30 minutes	-Keele STarT Back (SBST) low back pain screening tool -Fear Avoidance Beliefs Questionnaire (FABQ).	Subjective improvement in pain Pain reduction
Imai, et al., 2021 ^( 39 )^ ; Japan	Progressive physical exercises and Pain Neuroscience Education (PNE)	3-4 times per week for 6 months	20-minute stretching and 30-minute walks	-Widespread Pain Index (WPI) -EuroQol 5-Dimensions (EQ5D-L) -Presenteeism Scale (WLQ)	Reduced lower back pain Reduced presenteeism
Lopes, et al., 2019 ^( 40 )^ ; Brazil	Adapted mindfulness	8 sessions	60 minutes	-Nordic Musculoskeletal Questionnaire (NMQ) -Catastrophic Pain Scale -World Health Organization Quality of Life Scale (WHOQOL-BREF)	Less pain, anxiety and catastrophizing Improved quality of life perception
Morais, et al., 2023 ^( 41 )^ ; Brazil	Seed-based auriculotherapy	8 sessions	10-15 minutes	-Visual analog scale for pain -Brief pain inventory -Rolland-Morris Disability Questionnaire and Quality of Life SF-36	Reduced intensity of chronic spinal pain Improved quality of life perception
Soler-Font, et al., 2019 ^( 42 )^ ; Spain	Multifaceted intervention at three prevention levels: I. Participatory ergonomics: Reduction of exposure to biomechanical and psychosocial risk factors II. Health promotion: Nordic walking, Mediterranean healthy eating and Mindfulness course III. Case management: early detection of and MSDs and MSP; Multidisciplinary support for return-to-work	Variable, over 12 months	Variable depending on the component	-Adapted Standardized Nordic Questionnaire -Work role functioning was assessed using the Spanish version of the Work Role Functioning Questionnaire (WRFQ-SpV)	A reduction in pain was observed in the neck, shoulders and upper back Stable work functioning Decreased need for work rescheduling requests
Sezgin & Esin, 2018 ^( 43 )^ ; Turkey	An ergonomic risk management program was developed based on the PRECEDE-PROCEED model. 1) Video training and educational materials (brochures and CDs), complemented with preventive exercises 2) Personal interviews focused on identifying predisposing, reinforcing and facilitating factors for behavior change	26 follow-up weeks	Two weeks	-Musculoskeletal symptoms questionnaire -Ergonomic risk report form -Rapid Upper Limb Assessment (RULA) form	Reduction in MSP Increased exercise frequency

## Discussion

The study objective was to analyze the effectiveness of individual coping strategies and adaptations in the hospital work environment for Nursing professionals with MSP. Although this issue is widely recognized in the Nursing field^(9-10,15)^, the available robust scientific evidence remains limited. The existing research has primarily focused on prevalence, risk factors, causality and preventive interventions related to MSDs and MSP^(12)^. However, considering that living without pain is a universal right^(44)^, it is crucial to identify better ways to adapt to or alleviate Nursing professionals’ physical suffering, even when pain does not pose an immediate threat to life^(45)^. In this context, it is necessary to adopt effective coping strategies for MSP, along with workplace interventions and adaptations that address Nursing professionals’ needs. The studies analyzed in this review provide evidence regarding the effectiveness of interventions with the potential to prevent MSP progression among Nursing professionals in hospital settings. Despite the heterogeneity observed in the strategies, including the number and duration of sessions (which ranged from interventions with eight weekly sessions to a one-year program^(42)^), the results demonstrated a reduction in pain intensity, improvements in physical functionality and work performance, and an increase in quality of life indicators for Nursing professionals.

The findings are presented according to the analysis categories established for this study.

### Coping strategies or actions implemented by Nursing professionals with work-related MSP

It is noteworthy that the studies included did not detail the specific strategies used by Nursing professionals in their professional practice but demonstrated their effectiveness in addressing MSP. These strategies may represent viable options for professionals to apply. As evidenced in the RCTs, the individual/personal strategies adopted by Nursing professionals presented high methodological quality in most of the studies^(36-39,42)^, including the seed-based auriculotherapy study^(41)^, which was classified with five points on the Jadad scale. However, one study on an ergonomic risk management program showed low quality, with two points^(43)^. This indicates lack of methodological homogeneity across the studies.

Most of the studies included focused on reducing low back pain due to its epidemiological relevance^(3)^ and its high prevalence among Nursing professionals, which ranges between 44.4% and 90.2%^(46-49)^. This high prevalence exerts impacts on work productivity and daily activities^(50)^. The most effective strategies included therapeutic exercises focused on improving spinal stabilization^(36-37)^ and stretching combined with other actions^(38-39,43)^. These findings are consistent with the results of other systematic reviews, as reported by Belgian researchers^(51)^ that evaluated the efficacy of interventions to prevent and treat low back pain in nurses, as well as a research study conducted in Italy, which analyzed interventions aimed at preventing and reducing MSDs in healthcare professionals, including nurses^(15)^.

From a physiological perspective, therapeutic exercises target muscle weakness and limited joint mobility, which are factors that increase the risk of musculoskeletal symptoms^(52)^. Moreover, they impose physical stress on the musculoskeletal system, thereby increasing the likelihood of overload-related injuries. In addition, low physical capacity self-assessments have been shown to predict low back pain in female healthcare workers^(52)^. Consequently, it is important to promote physical activity and exercise as both preventive strategies and therapeutic self-care approaches for Nursing professionals with MSP.

The interventions that combined physical exercise with other actions demonstrated effectiveness in reducing the MSP levels. Among them, the use of both physical and psychological approaches was identified^(36-39,42-43)^. These interventions combined physical exercise with psychosocial components, which also demonstrated self-reported reductions in fear of pain, anxiety and stress^(38-39)^. The evidence supports the effectiveness of these interventions, particularly those that incorporate psychosocial aspects, which are more effective than usual care or physical treatments alone^(53)^. This finding should be considered by Nursing professionals and employers alike, not only as individual therapy but also as a preventive measure against MSP. Characterized by high demands, low control and low social support, psychological stress has been associated with presence of MSP (OR=1.81; 95% CI: 1.50-2.18)^(18)^. Furthermore, studies such as the one conducted in Mumbai, India, have reported a similar relationship between workplace stress among Nursing professionals and other musculoskeletal disorders^(54)^. It is important to note that psychosocial factors in the work environment can act as stressors, triggering physiological stress responses, including biochemical processes that cause short-term muscle tension and increased MSP risk in the long term^(53)^. The way in which these adverse factors affect each person may vary depending on their individual characteristics, the available resources and the coping strategies employed. Therefore, it is essential for Nursing professionals to address both work-related stress and personal stress, as both are risk factors in MSP management^(55)^.

The mindfulness-based therapeutic program^(40)^ adapted from Mindfulness-Based Stress Reduction (MBSR)^(56)^ significantly reduced pain, as well as the musculoskeletal symptoms and anxiety/depression levels (p<0.001). Some studies support its effectiveness and safety in reducing chronic low back pain intensity in adults, including reduced analgesic use, as also found in other literature review studies^(57-58)^. Despite these results, its application in the practice remains poorly understood.

Seed-based auriculotherapy^(41)^ showed positive effects in reducing pain intensity among healthcare workers with chronic pain. No other similar studies were found during the review. This therapy remains underexplored in the Americas. A study conducted in Korea^(59)^ revealed that auriculotherapy significantly reduced MSP in adults. Furthermore, the longer the intervention period, the greater the observed effect size. This therapy has also demonstrated positive results in mental health care, alleviating stress, anxiety and depression^(60)^. Similarly, a study conducted in perioperative units demonstrated the benefits of auriculotherapy in relieving anxiety and stress among Nursing professionals^(61)^. Mindfulness and seed-based auriculotherapy should be options for MSP self-management in Nursing staff, and their use should be promoted in clinical settings.

It should be noted that the review identified studies that did not meet the inclusion criteria; however, they referenced actions performed by Nursing professionals to alleviate MSP and that deserve to highlighted, such as analgesic use^(16,62)^; self-medication (use of allopathic and homeopathic medications), which has been documented to have a prevalence ranging from 30% to 85.6%^(8,17,48,63-65)^; rest and physiotherapy^(62)^; correct posture in patient care; maintaining greater attention in all activities to be performed; performing functions with more calm and time; and participating in training offered by the institutions^(66)^, in addition to other complementary therapies that have been reported: aromatherapy, acupuncture, chiropractic, *reiki* and acupressure^(17,67)^. Alternative therapies that have been tested or applied in other populations should be considered and subjected to verification by higher-quality studies in Nursing professionals.

### Interventions and/or strategies to promote adaptation of Nursing professionals with MSP implemented and/or adopted in the work environment

The scientific evidence highlights that multidimensional interventions encompassing all three prevention levels (primary, secondary and tertiary) are more effective due to the multifactorial nature of work-related MSP^(51)^. These interventions align with occupational health organization guidelines, which recommend implementing multidisciplinary and integrated preventive strategies to address MSDs^(24,30)^. Therefore, it is essential that institutions consider these interventions and evaluate their effectiveness according to pain type, avoiding limiting themselves solely to physical treatments for pain reduction or granting disability leaves to Nursing professionals.

The study conducted with hospital nurses in Spain^(42)^ included components from all three prevention levels during 12 follow-up months; its results demonstrated a 63% reduction in neck, shoulder and upper back pain risk, but showed no statistical significance in the case of low back pain. In turn, studies show that multidisciplinary biopsychosocial interventions are marginally more effective for chronic low back pain rehabilitation^(53)^.

Another study focused on workplace ergonomics, addressing ergonomic risk diagnosis^(43)^; although its methodological quality and bias risk limit reproducibility of its results, it is important to note that it demonstrated that training in musculoskeletal risk identification and prevention and applied training in risks during patient mobilization and transfer yielded favorable results in reducing MSP intensity, in line with recommendations from other interventions^(42,68)^ and occupational organizations^(24)^, which agree that ergonomic training and workplace adjustments can reduce ergonomic risk, improve musculoskeletal symptoms and enhance productivity. In contrast, they do not recommend combining them with occupational stress management training, as no significant effect was found^(69)^.

Although the results are positive, it is important to consider that ergonomics focuses on adapting working conditions and identifying risks, being only an indicator of a larger workplace problem^(20)^.

Other strategies described in studies not included in this review report that adjusting work schedules can reduce shoulder pain risk. Avoiding excessively prolonged work shifts (more than 46 hours a week) can prevent shoulder and neck pain^(70)^. Availability and use of mechanical equipment for patient mobilization and transfer are associated with reduced shoulder pain risk^(71)^. Some systematic reviews^(15,72)^ have reported that using mechanical or sliding lifts is related to reduced prevalence of work-related injuries and pain among healthcare professionals, including Nursing professionals. Other studies^(2,73-74)^ indicate that factors such as discontinuous/night shifts and working in areas such as operating rooms, surgical services, emergency rooms, intensive care units, Gynecology and Obstetrics^(75-78)^ have been associated with increased MSP risks. Therefore, managers should consider these variables when planning service and shift distribution, adapting them to individual needs of the staff with MSP.

In this research, no experimental studies were retrieved that would support the effect of ergonomic adaptations, as well as implementations or changes in infrastructure, physical space improvements, ergonomic furniture, staffing strategies, service organization or workplace processes. These interventions might contribute to reducing MSDs and MSP, as well as ease rehabilitation or ensure safe return to work activities^(24,79-80)^. The absence of solid evidence may be due to associated costs and hospital organizational management policies. Nevertheless, it is relevant to consider these needs to provide comprehensive care in the Nursing staff work environment and adopt more effective intervention strategies.

The importance of implementing effective interventions in the hospital work environment is highlighted to prevent Nursing professionals from developing MSDs, as well as to ensure that those already presenting MSP can continue working under dignified conditions, in an environment adapted to their physical and psychological conditions.

Limitations: the limited number of experimental and pre-experimental studies on the topic, methodology quality, small sample sizes, number of studies with bias risk and number of implemented interventions were limiting factors for a deeper analysis of the results, which precluded performing a meta-analysis. Due to the specificity of the Nursing population in identification of the studies, some studies conducted with other healthcare professionals might have been omitted, including Nursing professionals.

## Conclusion

This review demonstrated that the most effective strategies are those that combine individual actions by Nursing professionals and improvements in working conditions considering physical, psychosocial and ergonomic aspects for work environment adaptations, preventing musculoskeletal pain progression and maintaining work performance.

The most effective strategies for addressing MSP in hospital Nursing professionals are physical exercise, stretching, auriculotherapy and mindfulness. It is fundamental to promote these self-care strategies, particularly in hospital environments with prolonged shifts and in services that generate greater physical and emotional burden. The most effective strategies related to the hospital work environment are those that adopt a comprehensive approach, combining physical, psychosocial and ergonomic aspects directed at employees and employers alike. This can prevent MSP progression and allows professionals to continue performing their work activities.

For future perspectives, it is crucial to conduct RCTs that comprehensively evaluate strategies aimed at individual needs, alternative therapies and specific workplace adaptations for the sake of Nursing professionals with MSP.

## Data Availability

All data generated or analysed during this study are included in this published article.
