# Supplementary material for: Adaptation strategies for nurses with musculoskeletal pain in hospital work: a systematic review
Source: Rev Lat Am Enfermagem. 2025 Nov 10;33:e4725. doi: 10.1590/1518-8345.7812.4725 (PMC12610933; doi:10.1590/1518-8345.7812.4725)
Supplement: Translation

# Translation: Estratégias de adaptação dos profissionais de enfermagem com dor musculoesquelética no trabalho hospitalar: uma revisão sistemática

**DOI:** 10.1590/1518-8345.7812.4726

**Authors:** Jorge Gabriel Tuz-Colli, Yolanda Flores-Peña, Heloisa Ehmke Cardoso dos Santos, Fernanda Ludmilla Rossi Rocha, Maria Helena Palucci Marziale

**Affiliations:** 1 Universidad Autónoma de Nuevo León, Facultad de Enfermería de Nuevo León, Monterrey, Nuevo León, México. Universidad Autónoma de Nuevo León Facultad de Enfermería de Nuevo León Nuevo León Monterrey México; 2 Universidade de São Paulo, Escola de Enfermagem de Ribeirão Preto, Centro Colaborador da OPAS/OMS para o Desenvolvimento da Pesquisa em Enfermagem, Ribeirão Preto, SP, Brasil. Universidade de São Paulo Escola de Enfermagem de Ribeirão Preto Centro Colaborador da OPAS/OMS para o Desenvolvimento da Pesquisa em Enfermagem SP Ribeirão Preto Brasil

**Keywords:** Dor Musculoesquelética, Habilidades de Enfrentamento, Condições de Trabalho, Ergonomia, Profissionais de Enfermagem, Enfermagem

## Abstract

analisar a efetividade das estratégias de enfrentamento individual e de adaptação do ambiente laboral hospitalar para os profissionais de enfermagem com dor musculoesquelética.

revisão sistemática conduzida de acordo com os *Preferred Reporting Items for Systematic reviews and Meta-Analyses*. A busca foi realizada em seis bases de dados, incluindo estudos experimentais e pré-experimentais. O risco de viés foi avaliado por meio das ferramentas RoB2 (*Risk of Bias 2*) e ROBINS-I (*Risk of Bias In Non-Randomized Studies of Interventions*). A qualidade metodológica foi avaliada pelo modelo *Joanna Briggs Institute*, Escala de Jadad e Nível de Evidência Melnyk, Buck e Gallagher-Ford.

foram incluídos oito estudos, identificando-se as estratégias de enfrentamento individual: exercícios de alongamento, auriculoterapia, *mindfulness*. As estratégias aplicadas no ambiente laboral são multidisciplinares, integradas e orientadas para a capacitação na identificação e prevenção de riscos musculoesqueléticos e para o aprimoramento das condições do ambiente de trabalho.

as estratégias mais efetivas são aquelas que combinam ações individuais dos trabalhadores com melhoria das condições de trabalho considerando aspectos físicos, psicossociais e ergonômicos para a adaptação do ambiente de trabalho, prevenir a progressão da dor musculoesquelética e manter o desempenho no trabalho. Registro PROSPERO CRD42024575014.

## Destaques

**(1)** Estratégias individuais e focadas no local de trabalho têm sido aplicadas para reduzir a DME. **(2)** Estratégias não farmacológicas como *mindfulness* e auriculoterapia reduzem a DME. **(3)** A aplicação de ergonomia no trabalho reduz a DME. **(4)** As necessidades individuais do trabalhador devem ser consideradas para reduzir a DME.

## Introdução

A dor musculoesquelética (DME) compromete ossos, músculos, ligamentos, tendões e nervos, constituindo o principal sintoma das doenças musculoesqueléticas (DoMEs). Manifesta-se com maior prevalência nas costas, pescoço e ombros, associando-se a doenças como osteoartrite e artrite reumatoide, bem como à dor decorrente de distensões musculares, fraturas, traumatismos, entre outros^(1-2)^.

As DoMEs acompanhadas de DME representam um grave problema de saúde pública que afeta a população trabalhadora, especialmente os profissionais de saúde, com maior prevalência entre os profissionais de enfermagem. Essas doenças constituem a principal causa de absenteísmo, limitação das capacidades funcionais e incapacidade permanente nesse grupo profissional. Além disso, costumam provocar diminuição da mobilidade e destreza, reduzindo a capacidade laboral, aumentando o absenteísmo, as aposentadorias precoces, elevando os custos da assistência à saúde, comprometendo a segurança dos pacientes e diminuindo a qualidade de vida^(3-5)^.

Entende-se por “profissionais de enfermagem” o conjunto de pessoas qualificadas e autorizadas a exercer a enfermagem, incluindo enfermeiros, técnicos, auxiliares e pessoal de apoio, conforme a regulamentação de cada país. Esse grupo representa aproximadamente 59% do total de trabalhadores da saúde, sendo o mais numeroso nos hospitais^(6)^. Devido à sua alta representatividade, à diversidade e grau de exigência de suas funções, e à constante exposição a riscos ocupacionais, como o manuseio de pacientes, transporte de objetos pesados e participação em procedimentos clínicos, os profissionais de enfermagem hospitalar encontra-se vulnerável ao desenvolvimento de DoMEs^(7)^.

Um estudo multicêntrico realizado em países de economias emergentes^(8)^ revelou prevalência de DoMEs de 37% na população trabalhadora, elevando-se para 92% entre profissionais de enfermagem hospitalares. Estudos de metanálise^(9)^ indicam alta prevalência de 64% na região lombar e 40% em pescoço e ombros^(10)^. Além disso, 33% dos afastamentos laborais nesse grupo estão relacionados às DoMEs^(10)^. A dor lombar se destaca como o principal fator contribuinte para a carga global desses distúrbios e representa a causa mais comum de incapacidade em 160 países^(3,11)^.

A prevenção das DoMEs relacionadas ao trabalho é muito importante e deve ser objeto de ações preventivas no ambiente laboral dos profissionais de enfermagem, assim como o uso de estratégias de adaptação para que o trabalhador que adoece por DoMEs e com dor musculoesquelética (DME) possa permanecer ativo. Enfrentar a dor é essencial para a qualidade de vida e as atividades da vida diária, no entanto, a pesquisa sobre o tema tem se concentrado no estudo da prevalência da DME, estudo dos fatores de risco (psicossociais, demográficos, físicos e mentais) e em intervenções direcionadas à prevenção do problema cuja implementação na prática não tem sido ótima^(12)^, dada a alta prevalência de DME a nível mundial, inclusive em países com economias de alta renda^(13-15)^.

Os poucos estudos sobre as estratégias de enfrentamento da DME em profissionais de enfermagem apontam o uso de diversas estratégias, algumas inclusive não recomendáveis. Entre as estratégias mais utilizadas, destacam-se o uso de analgésicos como Paracetamol e anti-inflamatórios não esteroidais (AINEs). Outras estratégias incluem ignorar a dor, buscar consulta médica com especialistas, recorrer a medicamentos alternativos, terapias complementares como massagens terapêuticas, aromaterapia, acupuntura, quiropraxia e *reiki*; autocontrole da dor, repouso na cama; fisioterapia e exercícios para o manejo da dor^(8,16-17)^.

Pesquisas recentes têm destacado a relação entre fatores individuais, psicossociais e a ergonomia com o desenvolvimento de danos musculoesqueléticos relacionados ao trabalho. Essa descoberta contrasta com a consideração tradicional que atribuía essas lesões principalmente à exposição a fatores de natureza biomecânica^(18-20)^.

A ergonomia busca adaptar o ambiente de trabalho às necessidades do trabalhador^(21-22)^. No ambiente hospitalar, a aplicação dos princípios da ergonomia pode prevenir o surgimento e a progressão das DoMEs e da DME, assim como melhorar a segurança, o estado de saúde e, consequentemente, a qualidade de vida no trabalho para os profissionais de enfermagem^(19,20,23)^.

Enfrentar a DME é essencial para preservar a produtividade dos profissionais de enfermagem, cujo desempenho não apenas representa seu sustento econômico, mas também uma força de trabalho necessária para atender à crescente demanda por serviços de saúde. Isso ganha relevância diante da escassez global de 5,9 milhões de profissionais de enfermagem, principalmente em regiões como África, Sudeste Asiático e Mediterrâneo Oriental e em países da América Latina, como República Dominicana, Honduras, Guiana e Venezuela, conforme informado pela Organização Pan-Americana da Saúde (OPAS) em 2020^(6)^.

Para aliviar a DME, os profissionais de enfermagem empregam estratégias de enfrentamento tanto individuais quanto no local de trabalho, com o objetivo de manter seu desempenho e prevenir a progressão do problema. No entanto, uma recuperação completa e reintegração segura ao trabalho requerem que empregadores e gestores implementem estratégias como o fornecimento de equipamentos para o transporte de pacientes e a implementação de horários flexíveis, adaptando as condições de trabalho às necessidades e capacidades do trabalhador (aplicação da ergonomia). Essas adaptações reduzem o risco de exacerbações e acidentes, especialmente em trabalhadores com capacidade funcional reduzida^(24)^.

No entanto, de acordo com a revisão bibliográfica, verificamos que a informação disponível sobre as estratégias de enfrentamento individual, assim como de adaptação no local de trabalho em profissionais de enfermagem é limitada. Portanto, realizamos esta revisão sistemática com o objetivo de analisar a efetividade das estratégias de enfrentamento individual e de adaptação do ambiente de trabalho hospitalar para os profissionais de enfermagem com dor musculoesquelética.

## Método

Revisão sistemática da literatura registrada na plataforma *International Prospective Register of Systematic Reviews* (PROSPERO), Número de registro CRD42024575014 em 16 de agosto de 2024, desenvolvida de acordo com as recomendações do guia para revisões do *Centre for Reviews and Dissemination* (CRD)^(25)^ dos *Preferred Reporting Items for Systematic reviews and Meta-Analyses* (PRISMA)^(26)^. Seguindo as etapas: a) Definição da pergunta de pesquisa, b) Protocolo de revisão: critérios de exclusão e inclusão de estudos, c) Busca bibliográfica: desenvolvimento da estratégia de busca, d) Seleção de estudos, e) Extração dos dados, f) Avaliação crítica da qualidade dos estudos incluídos, g) Síntese dos resultados e h) Apresentação e divulgação dos resultados.

### Definição da pergunta de pesquisa

A pergunta de pesquisa foi a seguinte: “Qual a efetividade das estratégias de enfrentamento individual e de adaptação do ambiente de trabalho hospitalar para os profissionais de enfermagem com dor musculoesquelética?”. A pergunta foi estruturada com base na estratégia PICOS^(27)^ (acrônimo de *Population*, *Interventions*, *Comparison*, *Outcome*, *Study design*), onde P (Participantes): profissionais de enfermagem que trabalham em hospitais (enfermeiros, técnicos e auxiliares de enfermagem) com DME; I (Intervenção): estratégias de enfrentamento individual e no ambiente de trabalho para a adaptação dos profissionais de enfermagem com DME; C (Comparação/Controle): não se aplicou; O (*Outcome*/Resultado): adaptação dos profissionais de enfermagem no ambiente de trabalho.

Definições: a) Profissionais de enfermagem: enfermeiros, técnicos de enfermagem, auxiliares de enfermagem. b) DME: experiência sensorial e emocional desagradável que afeta ossos, músculos, ligamentos, tendões e nervos, sendo o principal sintoma nas DoMEs.

### Protocolo de revisão: critérios de inclusão e de exclusão

Critérios de inclusão: Estudos primários que respondem à pergunta de investigação sobre a efetividade das estratégias de enfrentamento individual e de adaptação aplicadas no ambiente de trabalho para os profissionais de enfermagem com DME. Foram incluídos estudos de desenho experimental (ensaios clínicos randomizados - ECR, ensaios clínicos - EC) e estudos pré-experimentais (pré e pós-teste com um grupo), artigos sem restrição de idioma, publicados entre janeiro de 2000 e julho de 2024.

Critérios de exclusão: Estudos que não respondiam à pergunta de investigação, profissionais de enfermagem que não trabalhavam em hospitais, estudos-piloto, descritivos, observacionais e revisões bibliográficas de qualquer tipo.

### Busca bibliográfica: desenvolvimento da estratégia de busca

As buscas foram realizadas nas seguintes bases de dados: MEDLINE/*PubMed*^®^, *Web of Science*, *Scopus*, *Cochrane Library*, *Cumulative Index to Nursing and Allied Health Literature* (CINAHL) e Literatura Latino-Americana e do Caribe em Ciências da Saúde (LILACS). A estratégia de busca foi desenvolvida mediante combinação de descritores controlados e palavras-chave, utilizando os operadores booleanos “*OR*” entre sinônimos e similares e “AND” entre palavras-chave, conforme as particularidades de cada base de dados. O desenvolvimento da busca contou com assessoria técnica de uma bibliotecária. A estratégia foi estruturada utilizando o vocabulário controlado do *Medical Subject Headings* (MeSH) e dos Descritores em Ciências da Saúde (DeCS). A busca nas bases de dados foi realizada entre 31 de agosto e 7 de setembro de 2024, incluindo artigos publicados entre janeiro de 2000 e julho de 2024. As referências foram gerenciadas no *software* Mendeley. A Figura 1 ilustra a estratégia de busca.

**Figura 1-:** Estratégias de busca. Ribeirão Preto, SP, Brasil, 2024

*PubMed* /MEDLINE (“Nursing Assistant*” OR “Personnel nursing” OR Nurse* OR “nursing worker*” OR “nursing professional*”) AND (“Musculoskeletal Pain”[Mesh] OR “Musculoskeletal Pain” OR “muscle pain”)
*Web of Science* (“Nursing Assistant*” OR “Personnel nursing” OR Nurse* OR “nursing worker*” OR “nursing professional*”) AND (“Musculoskeletal Pain” OR “muscle pain”)
*Scopus* (“Nursing Assistant*” OR “Personnel nursing” OR Nurse* OR “nursing worker*” OR “nursing professional*”) AND (“Musculoskeletal Pain” OR “muscle pain”)
*Cochrane* ((MeSH descriptor: [Nursing NEXT Assistant*] explode all trees) OR (Personnel NEXT nursing) OR Nurse OR Nurses OR (nursing NEXT worker*) OR (nursing NEXT professional*)) AND ((MeSH descriptor: [Musculoskeletal Pain] explode all trees) OR “Musculoskeletal Pain” OR “muscle pain”)
CINAHL (“Nursing Assistant*” OR “Personnel nursing” OR Nurse* OR “nursing worker*” OR “nursing professional*”) AND ((MH “Musculoskeletal Pain”) OR “Musculoskeletal Pain” OR “muscle pain”)
BVS – LILACS (“Profissional de enfermagem” OR “Profissionais de enfermagem” OR “Profissionais da enfermagem” OR “Técnico de enfermagem” OR “trabalhadores da enfermagem” OR “trabalhador da enfermagem” OR “trabalhador de enfermagem” OR “trabajadores de enfermería” OR “trabajador de enfermería” OR “Assistentes de Enfermagem” OR “Auxiliar de Enfermagem” OR “Auxiliar do Enfermeiro” OR “Auxiliares de Enfermagem” OR “Auxiliares de Enfermeiros” OR “Asistentes de Enfermería” OR “asistente de enfermería” OR “auxiliar de enfermería” OR “auxiliar del enfermero” OR “auxiliares de enfermeros” OR “auxiliares de enfermería” OR enfermeri* OR Enfermero* OR Enfermera* OR “Healthcare Assistant*” OR “Nursing Assistant*” OR “Personnel nursing” OR “nursing professional* OR Nurse* OR”nursing worker*” OR “nursing professional*” OR Enfermagem OR Enfermería OR Nursing) AND (“Dor Musculoesquelética*” OR “Dores Musculoesqueléticas*” OR “Dor muscular*” OR “Dores musculares” OR “Dor Osteomuscular*” OR “dolor osteomuscular*” OR “Dolor Musculoesquelético” OR “Musculoskeletal Pain” OR “muscle pain”)

### Seleção de estudos

Os resultados da busca nas bases de dados foram exportados para o *software* online *Rayyan*^®(28)^, utilizado para armazenar, organizar, eliminar duplicatas e selecionar os estudos de forma cega. Os estudos foram selecionados em duas fases. A primeira mediante análise dos títulos e resumos, realizada por dois revisores de forma independente e anônima, seguindo os passos do diagrama de fluxo do PRISMA^(26)^. Na segunda fase, foram analisados os artigos em texto completo selecionados e aplicados os critérios de inclusão, de forma mascarada (revisores R1 e R2) com a finalidade de limitar o viés na revisão (setembro 2024). A resolução de conflitos por discrepâncias dos estudos ficou a cargo de outro pesquisador especialista no tema (R3) através da discussão dos critérios de seleção.

Os revisores empregaram as seguintes definições para orientar as decisões:

Enfrentamento individual de trabalhadores com DME: são as ações cognitivas e comportamentais dinâmicas que os profissionais de enfermagem desenvolvem para enfrentar situações estressantes, aliviar e enfrentar a DME para manter suas atividades no local de trabalho^(29)^.

Adaptação do ambiente de trabalho para trabalhadores com DME: alcançada mediante intervenções no local de trabalho (ambiente de trabalho) como atividades, tarefas e produtos desenvolvidos ou implementados para alterar ou reduzir a ação dos riscos ocupacionais e incrementar os conhecimentos, comportamentos e atitudes dos trabalhadores. Essas intervenções podem ser desenvolvidas diretamente no ambiente de trabalho ou direcionadas aos trabalhadores, através de educação, formação continuada e adequação do ambiente de trabalho às características psicofisiológicas dos profissionais de enfermagem^(30)^.

### Extração dos dados

Para a extração de dados dos estudos selecionados, foi empregada uma tabela de caracterização elaborada em *Microsoft Excel*^®^ que incluiu: ano de publicação, país de origem, objetivo, tipo de desenho do estudo, critérios de inclusão e exclusão da amostra, detalhes do tipo e método de análise dos dados, descrição da intervenção, instrumento de avaliação, resultados, conclusões e nível de evidência.

Os achados de maior relevância e as conclusões dos estudos selecionados, relacionados com a pergunta de revisão foram analisados a partir de duas categorias prévias:

Intervenções e/ou estratégias de enfrentamento utilizadas pelos profissionais de enfermagem para lidar com a DME relacionada ao trabalho.

Intervenções e/ou estratégias para promover a adaptação dos profissionais de enfermagem com DME, realizadas e/ou adotadas no ambiente de trabalho.

### Avaliação crítica da qualidade dos estudos

Foram utilizadas ferramentas para avaliar a qualidade metodológica dos estudos passíveis de revisão, considerando tanto o método utilizado quanto o conteúdo, para isso aplicamos as ferramentas de avaliação crítica do *Joanna Briggs Institute* (JBI)^(31)^. Para a análise da qualidade metodológica dos ensaios clínicos randomizados (ECR) foi utilizada a escala de Jadad^(32)^, que avalia três itens do estudo através de cinco perguntas binárias com respostas “Sim” (vale 1) ou “Não” (vale 0). Os itens analisam a randomização, o mascaramento, as perdas e abandonos de participantes; para uma pontuação geral de 0 a 5. Os resultados de 0-2 são considerados de “baixa qualidade metodológica” e 3-5 “alta qualidade metodológica”.

Para determinar o nível de evidência dos estudos selecionados, seguimos o marco estabelecido por Melnyk, Buck e Gallagher-Ford^(33)^. Este passo também foi realizado por revisores de forma independente e mascarada. O terceiro revisor foi chamado para resolver possíveis conflitos na avaliação.

### Avaliação do risco de viés

O risco de viés dos ensaios clínicos randomizados foi avaliado por dois revisores independentes mediante a ferramenta *Risk of Bias 2* (RoB2)^(34)^. Para os estudos clínicos não randomizados pré-experimentais, foi empregada a ferramenta *Risk of Bias In Non-Randomized Studies of Interventions* (ROBINS-I)^(35)^.

### Aspectos éticos

O estudo secundário não requer aprovação do Comitê de Ética em Pesquisa. Contudo, os autores declaram que não existem conflitos de interesses que comprometam a análise dos resultados deste trabalho.

## Resultados

Foram recuperados um total de 1.732 estudos: 316 (18,2%) artigos no *PubMed*/MEDLINE; 435 (25,1%) no *Scopus*, 417 (24,1%) na *Web of Science*; 281 (16,2%) na *Cochrane Library*; 213 (12,3%) no CINAHL e 70 (4%) no LILACS. Após a eliminação de registros duplicados (n=711), foram identificados 1.021 estudos para revisão de seus títulos e resumos. Restaram 33 publicações potenciais para revisão do artigo completo e, após a revisão do texto, 8 artigos científicos foram incluídos neste estudo.

A Figura 2 mostra o fluxograma da busca na literatura e dos critérios de seleção adaptado do PRISMA^(26)^.

**Figura 2-:** Fluxograma da busca na literatura e dos critérios de seleção adaptado do PRISMA^(26)^

Com relação às características do estudo, todos os artigos estavam publicados em língua inglesa; foram publicados 3 (37,5%) estudos no Brasil, 2 (25,0%) no Japão, 1 (12,5%) na Espanha, 1 (12,5%) na Finlândia e 1 (12,5%) na Turquia. Quanto à população estudada, em 4 (50,0%) estudos participaram enfermeiras e em 4 (50,0%) trabalhadores da saúde que incluíram principalmente enfermeiras.

### Síntese de evidências

A análise do nível de evidência dos estudos foi realizada através do marco estabelecido por Melnyk, Buck e Gallagher-Ford. Foram classificados no nível II 8 estudos experimentais: 7 ECR^(36–42)^ e 1 estudo pré-experimental^(43)^.

Na avaliação da qualidade metodológica dos sete ECR com base na escala de Jadad^(32)^, encontrou-se que 6 (85,7%) estudos foram de alta qualidade metodológica^(36-39,41-42)^ e um (14,30%) de baixa qualidade^(40)^. O estudo pré-experimental^(43)^ foi qualificado como de baixa qualidade metodológica.

Os instrumentos de avaliação da DME variaram segundo o estudo. Eles incluíram a Escala Visual Analógica (EVA), adaptações do questionário Nórdico de Sintomas Musculoesqueléticos e o Questionário de Crenças de Medo e Evitação (*Fear-Avoidance Beliefs Questionnaire*, FABQ).

Os estudos analisados avaliaram a efetividade de diversas intervenções implementadas em contextos hospitalares, direcionadas a profissionais de enfermagem com DME relacionada ao trabalho. As estratégias de enfrentamento individual reportadas incluíram: exercícios terapêuticos, Pilates, alongamentos ativos e progressivos^(36-39)^; terapias como o *mindfulness* adaptado^(40)^; auriculoterapia^(41)^ e educação em neurociência da dor^(39)^. As intervenções direcionadas a favorecer a adaptação no ambiente de trabalho compreenderam uma intervenção multifacetada com enfoque participativo nos três níveis de prevenção^(42)^ e um programa de gestão de riscos ergonômicos^(43)^.

A Figura 3 apresenta os resultados da avaliação do risco de viés nos estudos.

As Figuras 4 e 5 apresentam as características dos estudos incluídos na revisão.

**Figura 3-:** Avaliação de risco de viés dos estudos incluídos por meio das ferramentas RoB2 (*Risk of Bias 2*) e ROBINS-I (*Risk of Bias In Non-Randomized Studies of Interventions*). Ribeirão Preto, SP, Brasil, 2024

**Figura 4-:** Características dos estudos incluídos na revisão sobre as estratégias ou ações de enfrentamento realizadas pelos profissionais de enfermagem com DME relacionada ao trabalho (n = 8). Ribeirão Preto, SP, Brasil, 2024

Primeiro autor, ano, país	Desenho do estudo	Nível de evidência/ Qualidade metodológica	Participantes	Intervenção	Resultados
Moreira, et al., 2021 ^( 36 )^ ; Brasil	Experimental, ensaio clínico controlado randomizado, simples-cego, de grupos paralelos com ocultação da alocação	Nível II 3	GI*: 46 auxiliares de enfermagem; e 44 do GC ^†^	O programa foi aplicado duas vezes por semana, com 30 minutos por sessão. O treinamento foi composto por exercícios para a estabilização da coluna. Cada sessão constava de três etapas: 1) Cinco minutos de exercícios de aquecimento; 2) 20 minutos de exercícios terapêuticos; 3) Cinco minutos de alongamento dos músculos isquiotibiais e exercícios de resfriamento ao final da sessão.	Os participantes exibiram melhora na força dos músculos flexores do tronco com tamanho de efeito 0,77 ( *p* =0,002) e no controle dos sintomas da região lombar 6,25 ( *p* =0,002) após o programa de exercícios terapêuticos. A associação positiva entre a exposição ao exercício e a melhora dos sintomas foi 2,04 vezes maior para o GI* que o GC ^†^ (RP ^‡^ =2,04; IC ^§^ 95%: 1,82-2,72).
Taulaniemi,et al.,2020 ^( 37 )^ ; Finlândia	Experimental, ensaio clínico controlado randomizado de quatro braços	Nível II 3	GI*: 165 trabalhadoras da saúde e 54 GC ^†^ . 87% eram enfermeiras	Programa de exercícios tipo Pilates, centrado no controle da postura neutra da coluna, dividido em três etapas. Etapa 1. Aulas de exercício neuromuscular supervisionado. Etapas II e III: Uma aula supervisionada e uma sessão em casa com ajuda de um DVD ou um folheto elaborado para o estudo. Nesta etapa foi permitido fazer exercício em sessões grupais supervisionadas.	A prática de Pilates com boa aderência reduziu os níveis de crenças de evitação (FAB) do medo relacionado ao trabalho com a redução da intensidade da dor lombar ( *r* =0,16; *p* =0,05). Identificaram que as estratégias de motivação devem focar em pessoas com níveis baixos de educação, menor condição física e altos níveis de FAB para alcançar melhor aderência ao exercício.
Oka, et al., 2019 ^( 38 )^ ; Japão	Analítico, prospectivo, randomizado, de grupos paralelos e multicêntrico com avaliações centrais	Nível II 3	4767 enfermeiras No grupo A, 1799 No grupo B, 1420 e no Grupo C, 1548, em 12 hospitais	O grupo A foi o grupo controle. Os participantes dos grupos B e C receberam um manual de exercícios que descrevia como fazer o exercício de extensão de costas em pé “ *One Stretch* ”, além de um seminário de 30 minutos. No grupo B, os participantes receberam apenas o seminário de 30 minutos. No grupo C, foi utilizado uma abordagem física e psicológica para o tratamento da dor lombar.	O exercício de extensão de costas em pé “ *One Stretch* ” melhorou e preveniu eficazmente a dor lombar. As taxas de melhora nos grupos A, B e C foram de 13,3%, 23,5% e 22,6%, respectivamente. As taxas de piora da dor foram de 13,0%, 9,6% e 8,1%, que diminuíram à medida que aumentou o grau de intervenção ( *p* <0,001).
Imai, et al., 2021 ^( 39 )^ ; Japão	Experimental, ensaio controlado aleatorizado de grupos paralelos	Nível II 3	104 participantes 53 GI* e 51 GC ^†^ e trabalhadores de saúde incluindo enfermeiros	Consistiu em um plano de seis meses de exercícios progressivos e Educação em Neurociência da Dor (END). Com alongamentos de 20 minutos e caminhadas de aproximadamente 30 minutos, 3-4 vezes por semana. O grupo controle recebeu feedback geral após responder um questionário.	O estudo demonstrou que uma combinação de END e exercício proporcionou melhores resultados para os pacientes: menor presenteísmo ( *F* =12,87; *p* =<0,001; η2=0,94), menor intensidade da dor ( *F* =11,0; *p* =<0,001; η2=0,1), menor estresse físico e melhor estado psicológico e qualidade de vida.
Lopes, et al., 2019 ^( 40 )^ ; Brasil	Experimental, ensaio clínico, prospectivo, aberto, de medidas repetidas	Nível II 2	GI*: 64 técnicos de enfermagem Sem GC ^†^	Programa *mindfulness* adaptado com sessões semanais de 60 minutos, durante 8 semanas consecutivas. Os participantes foram orientados para uma prática diária de meditação em casa de 20 minutos, incluindo quando realizavam suas rotinas e atividades diárias como uma prática informal. Nos encontros presenciais, os exercícios focaram no manejo da dor, técnicas de respiração, escaneamento corporal, caminhada consciente, movimentos conscientes com posturas corporais leves, sentar, deitar e meditação.	Os resultados do programa *mindfulness* indicaram redução significativa dos sintomas musculoesqueléticos, dos níveis de ansiedade, depressão e dor catastrófica ( *p* <0,001). Um achado muito significativo foi a diminuição dos níveis de dor catastrófica (η2=0,203; *p* <0,001) e dor musculoesquelética (η2=0,200; *p* <0,001). Os efeitos positivos foram identificados após 8 semanas e se mantiveram inalterados até a semana 12 de seguimento.
Morais, et al., 2023 ^( 41 )^ ; Brasil	Experimental, Ensaio clínico randomizado, triplo cego (paciente, estatístico e avaliadores de resultados)	Nível II 5	GI* 34 trabalhadores; e 33 no GC ^†^ , em trabalhadores da saúde (incluindo pessoal de enfermagem) com diagnóstico de crônica na coluna vertebral	Ofereceram oito sessões de auriculoterapia com sementes, em duas aplicações por semana, com duração média de 10 a 15 minutos. Instruíram os trabalhadores a deixar por 3 dias com estimulação diária de forma manual pelo menos três vezes ao dia, 15 vezes em cada ponto auricular.	A auriculoterapia com sementes mostrou efeitos positivos como prática terapêutica na redução da intensidade da dor entre profissionais de saúde que apresentavam dor crônica na coluna vertebral, com redução significativa de 34% do GI* em relação ao GC ^†^ ( *p* =0,007). Quanto à interferência da dor nas atividades da vida diária, observou-se redução dos valores médios em ambos os grupos ( *p* <0,05). Também foi observada redução no uso de medicamentos ( *p* =0,013).
Soler-Font,et al.,2019 ^( 42 )^ ; Espanha	Experimental, ensaio clínico controlado randomizado por conglomerados de dois braços	Nível II 3	GI*: 138 enfermeiras; e 119 no GC ^†^	Utilizaram componentes nos três níveis de prevenção. Os componentes de prevenção primária foram: 1) fatores de risco ocupacional para proteger trabalhadores saudáveis das doenças musculoesqueléticas e ausências por doenças relacionadas a elas através da ergonomia participativa; e 2) promoção de estilos de vida saudáveis no trabalho (atividade física, bem-estar emocional e dieta). A prevenção secundária e terciária compreendia um serviço de gestão de casos para identificar dor musculoesquelética precocemente.	A intervenção foi efetiva para reduzir a dor em até 63% (RP ^‡^ =0,37; IC ^§^ 95%: 0,14-0,96) em pescoço, ombros e parte superior das costas no pessoal de enfermagem. Observou-se redução não estatisticamente significativa da dor lombar no grupo de intervenção comparado aos grupos controle. O funcionamento laboral manteve-se estável ao longo dos 12 meses de seguimento no grupo de intervenção comparado ao grupo controle ( *p* <0,05).
Sezgin & Esin, 2018 ^( 43 )^ ; Turquia	Pré-experimental Pré-teste e pós-teste para grupos controle não equivalentes	Nível II 2	61 enfermeiras: 30 do GI* e 31 do GC ^†^	O programa de gestão de riscos ergonômicos (PGRE) consistiu em duas intervenções: 1) capacitação em vídeo de duas semanas focada nos riscos musculoesqueléticos das enfermeiras de UTI, complementada com exercícios de prevenção; 2) entrevistas individuais com as enfermeiras focadas em identificar os fatores predisponentes, reforçadores e facilitadores da mudança de comportamento.	O PGRE foi eficaz para aumentar a frequência de exercício ( *p* =0,017) e diminuir a dor musculoesquelética ( *p* =0,017) e os níveis de risco ergonômico das enfermeiras de UTI ( *p* <0,05).

*GI = Grupo Intervenção; ^†^GC = Grupo Controle; ^‡^RP = Razão de Probabilidades; ^§^IC = Intervalo de Confiança

**Figura 5-:** Descrição das intervenções aplicadas nos estudos incluídos (n = 8). Ribeirão Preto, SP, Brasil, 2024

Primeiro autor, ano, país	Intervenção/Técnica usada	Número de sessões	Duração por sessão	Instrumento/Técnica de avaliação	Resultado
Moreira, et al., 2021 ^( 36 )^ ; Brasil	Exercício terapêutico para a força muscular da coluna (aquecimento, fortalecimento e alongamento)	17,5 sessões em média	30 minutos	Dinamometria: Força de músculos flexores do tronco; limiar de dor à pressão do dorsal longo	Maior força de flexores Diminuição significativa do limiar de dor por pressão
Taulaniemi, et al., 2020 ^( 37 )^ ; Finlândia	Exercício físico tipo Pilates Exercício neuromuscular	2 vezes por semana nos primeiros dois meses Posteriormente, 1 vez por semana por 4 meses de seguimento	60 minutos	- Questionário de crenças de evitação da dor (FABQ). - Registro de aderência ao exercício	Menor crença de evitação da dor relacionadas com a atividade física e o trabalho Maior aderência ao exercício
Oka, et al., 2019 ^( 38 )^ ; Japão	Exercícios de extensão ativa das costas “ *One Stretch* ” Seminário educativo Enfoques psicológicos e físicos	1 seminário e seguimento de 6 meses	30 minutos	- Ferramenta de detecção de dor lombar *Keele STarT Back* (SBST) - Questionário de crenças de evitação da dor (FABQ).	Melhoria subjetiva da dor Diminuição da dor
Imai, et al., 2021 ^( 39 )^ ; Japão	Exercícios físicos progressivos e Educação em Neurociência da Dor (END)	3-4 vezes por semana por 6 meses	Alongamentos de 20 minutos e caminhadas de 30 minutos	- Índice de dor generalizada (WPI) - EuroQol de 5 dimensões (EQ5D-L); - Escala de presenteísmo (WLQ)	Menor dor lombar Menos presenteísmo
Lopes, et al., 2019 ^( 40 )^ ; Brasil	*Mindfulness* adaptado	8 sessões	60 minutos	- Questionário Nórdico de Sintomas Musculoesqueléticos - Escala de dor catastrófica - Escala de Qualidade de vida da OMS (WHOQOL-BREF)	Menor dor, ansiedade e catastrofismo Maior percepção de qualidade de vida
Morais, et al., 2023 ^( 41 )^ ; Brasil	Auriculoterapia com sementes	8 sessões	10-15 minutos	- Escala visual analógica da dor -Inventário Breve da dor - Questionário de incapacidade de Rolland-Morris e de Qualidade de vida SF-36	Menor intensidade da dor crônica espinal Melhora da percepção de qualidade de vida
Soler-Font,et al.,2019 ^( 42 )^ ; Espanha	Intervenção multifacetada nos três níveis de prevenção: I. Ergonomia participativa: redução de exposição a fatores de risco biomecânico e psicossociais II. Promoção de saúde: caminhada nórdica, Alimentação saudável mediterrânea e Curso de *Mindfulness* III. Gestão de casos: detecção precoce de doenças musculoesqueléticas e dor musculoesquelética; Apoio para o retorno ao trabalho multidisciplinar	Variável, durante 12 meses	Variável dependendo do componente	- Questionário Nórdico Padronizado adaptado - Funcionamento laboral mediante o *Work Role Functioning Questionnaire* , versão em espanhol (WRFQ-SpV)	Menor dor no pescoço, ombros e parte alta das costas Funcionamento laboral estável Menor solicitação de reprogramação de trabalho
Sezgin & Esin, 2018 ^( 43 )^ ; Turquia	Programa de gestão de riscos ergonômicos baseado no modelo PRECEDE-PROCEDE: 1) Capacitação em vídeo e material educativo (folhetos e CD) complementado com exercícios de prevenção 2) Entrevistas pessoais focadas em identificar os fatores predisponentes, reforçadores e facilitadores da mudança de conduta	26 semanas de seguimento	Duas semanas	- Questionário de sintomas musculoesqueléticos - Formulário de relatório de riscos ergonômicos - Formulário de avaliação rápida de riscos superiores (RULA)’	Menor dor musculoesquelética Aumento da frequência de exercício

## Discussão

O objetivo do estudo foi analisar a efetividade das estratégias de enfrentamento individual e de adaptação do ambiente de trabalho hospitalar para os profissionais de enfermagem com DME. Embora este problema seja amplamente reconhecido no âmbito da enfermagem^(9-10,15)^, a evidência científica sólida disponível ainda é limitada. A pesquisa disponível tem se focado na prevalência, nos fatores de risco, na causalidade e nas intervenções preventivas relacionadas com as DoMEs e a DME^(12)^. No entanto, considerando que viver sem dor é um direito universal^(44)^, é crucial encontrar melhores formas de adaptação ou alívio do sofrimento físico dos profissionais de enfermagem, ainda que a dor não represente uma ameaça imediata à vida^(45)^. Neste contexto, é necessário adotar estratégias efetivas de enfrentamento da DME, juntamente com intervenções e adaptações no ambiente de trabalho que respondam às suas necessidades. Os estudos analisados nesta revisão fornecem evidência da efetividade das intervenções com potencial para prevenir a progressão da DME nos profissionais de enfermagem em ambientes hospitalares. Apesar da heterogeneidade observada nas estratégias, no número e duração das sessões que variaram desde intervenções de oito sessões por semana até um programa de um ano^(42)^, os resultados demonstraram diminuição na intensidade da dor, melhora na funcionalidade física, desempenho laboral e aumento nos indicadores de qualidade de vida dos profissionais de enfermagem.

Os resultados são apresentados de acordo com as categorias de análise estabelecidas para este estudo.

### Estratégias ou ações de enfrentamento realizadas pelos profissionais de enfermagem com DME relacionada ao trabalho

É importante destacar que os estudos incluídos não detalham as estratégias específicas utilizadas pelos profissionais de enfermagem em sua prática profissional, mas demonstraram efetividade para enfrentar a DME. Essas estratégias representam alternativas viáveis e fundamentadas para implementação pelos profissionais. As estratégias individuais/ pessoais adotadas pelos profissionais de enfermagem, evidenciadas nos ensaios clínicos randomizados, apresentaram elevada qualidade metodológica na maioria dos estudos analisados^(36-39,42)^, incluindo o estudo sobre auriculoterapia com sementes^(41)^, que foi classificado com cinco pontos na escala de Jadad. Contudo, um estudo específico sobre programa de gestão de riscos ergonômicos demonstrou baixa qualidade metodológica, obtendo apenas dois pontos^(43)^. Essa variação indica uma significativa falta de homogeneidade metodológica entre os estudos revisados.

A maioria dos estudos incluídos concentrou-se na redução da dor lombar devido à sua relevância epidemiológica^(3)^ e alta prevalência nos profissionais de enfermagem, que oscila entre 44,4% e 90,2%^(46-49)^. Esta elevada prevalência impacta a produtividade laboral e as atividades diárias^(50)^. As estratégias mais efetivas incluíram exercícios terapêuticos focados na melhoria da estabilização da coluna vertebral^(36-37)^ e alongamentos combinados com outras ações^(38-39,43)^. Esses achados são consistentes com os resultados de outras revisões sistemáticas, conforme relatado por pesquisadores belgas^(51)^, que avaliaram a eficácia de intervenções para prevenir e tratar a dor lombar em enfermeiros. Os resultados também se alinham com uma pesquisa realizada na Itália, que analisou intervenções voltadas para a prevenção e redução de distúrbios musculoesqueléticos em profissionais de saúde, incluindo os de enfermagem^(15)^.

Sob uma perspectiva fisiológica, os exercícios terapêuticos abordam a fraqueza muscular e a mobilidade articular limitada, fatores que aumentam o risco de sintomas musculoesqueléticos^(52)^. Além disso, esses fatores produzem sobrecarga física no sistema musculoesquelético, o que aumenta a probabilidade de lesões por sobrecarga. Também foi demonstrado que autoavaliações de baixa capacidade física são capazes de predizer a dor lombar em trabalhadoras da área da saúde^(52)^. Consequentemente, é importante promover a atividade física e o exercício como estratégias de prevenção e autocuidado terapêutico dos profissionais de enfermagem com DME.

As intervenções que combinaram exercício com outras ações demonstraram ser eficazes na redução dos níveis de DME. Entre elas, identificou-se o uso de abordagens tanto físicas quanto psicológicas^(36-39,42-43)^. Essas intervenções combinaram exercício físico com componentes psicossociais que também mostraram redução autorrelatada de medo da dor, ansiedade e estresse^(38-39)^. A evidência sustenta a efetividade dessas intervenções, destacando que aquelas que incorporam aspectos psicossociais demonstram maior eficácia em comparação às de atenção habitual ou aos tratamentos físicos^(53)^. Este achado deve ser considerado tanto pelos profissionais de enfermagem quanto pelos empregadores, não apenas como terapia individual, mas também como estratégia preventiva para a DME. O estresse psicológico, caracterizado por altas demandas, baixo controle e baixo apoio social, tem sido associado à presença de DME (RP=1,81; IC 95%: 1,50-2,18)^(18)^. Além disso, estudos como o realizado em Mumbai, na Índia, têm reportado relação similar entre o estresse laboral em profissionais de enfermagem e outras morbidades musculoesqueléticas^(54)^. Cabe destacar que os fatores psicossociais no ambiente de trabalho podem atuar como estressores, desencadeando reações fisiológicas por estresse, incluindo processos bioquímicos que geram tensão muscular a curto prazo e, a longo prazo, aumentam o risco de DME^(53)^. A forma como esses fatores adversos afetam cada indivíduo pode variar dependendo de suas características individuais, dos recursos disponíveis e das estratégias de enfrentamento utilizadas. Portanto, para os profissionais de enfermagem é fundamental abordar tanto o estresse relacionado ao trabalho quanto o estresse pessoal, uma vez que ambos constituem fatores de risco no controle da DME^(55)^.

O programa terapêutico baseado em *Mindfulness*^(40)^, adaptado do *Mindfulness-Based Stress Reduction* (MBSR)^(56)^, reduziu significativamente a dor e também os sintomas musculoesqueléticos, os níveis de ansiedade e depressão (*p*<0,001). Estudos comprovam sua eficácia e segurança para diminuir a intensidade da dor lombar crônica em adultos, incluindo a redução do uso de analgésicos, conforme também encontrado em outras revisões da literatura^(57-58)^. Apesar dos resultados promissores, sua aplicação na prática clínica ainda é pouco conhecida.

A auriculoterapia com sementes^(41)^ apresentou efeitos positivos na redução da intensidade da dor entre profissionais de saúde com dor crônica. Durante a revisão, não foram encontrados outros estudos similares. Trata-se de uma terapia pouco explorada no continente americano. Um estudo realizado na Coreia^(59)^ revelou que a auriculoterapia diminuiu significativamente a DME em adultos. Além disso, quanto mais prolongado o período de intervenção, maior foi o tamanho do efeito observado. Esta terapia também demonstrou resultados positivos na atenção à saúde mental, aliviando o estresse, a ansiedade e a depressão^(60)^. Da mesma forma, um estudo realizado em unidades perioperatórias demonstrou os benefícios da auriculoterapia no alívio da ansiedade e do estresse entre profissionais de enfermagem^(61)^. O *Mindfulness* e a auriculoterapia com sementes devem ser considerados opções para o autocontrole da DME para os profissionais de enfermagem, sendo necessário fomentar seu uso em ambientes clínicos.

Vale destacar que a revisão identificou estudos que não atendiam aos critérios de inclusão, mas que relatavam ações realizadas pelos profissionais de enfermagem para aliviar os distúrbios musculoesqueléticos e merecem ser mencionadas. Entre essas ações estão: o uso de analgésicos^(16,62)^; a automedicação (com medicamentos alopáticos e homeopáticos), cuja prevalência documentada varia entre 30% e 85,6%^(8,17,48,63-65)^; o repouso e a fisioterapia^(62)^; a adoção de posturas corretas durante o cuidado ao paciente; maior atenção na execução de todas as atividades; a realização das tarefas com mais calma e tempo adequado; e a participação em capacitações oferecidas pela instituição^(66)^. Além disso, outras terapias complementares relatadas incluem: aromaterapia, acupuntura, quiropraxia, *reiki* e acupressão^(17,67)^. As terapias alternativas já testadas em outras populações ou aplicadas devem ser consideradas e avaliadas por meio de estudos de maior qualidade voltados aos profissionais de enfermagem.

### Intervenções e estratégias para promover a adaptação dos profissionais de enfermagem com DME implementadas no ambiente de trabalho

As evidências científicas demonstram que intervenções multidimensionais, abrangendo os três níveis de prevenção (primária, secundária e terciária), são mais eficazes devido à natureza multifatorial dos distúrbios musculoesqueléticos relacionados ao trabalho^(51)^. Essas intervenções estão alinhadas com as diretrizes de organizações de saúde ocupacional, as quais recomendam a implementação de estratégias preventivas multidisciplinares e integradas para o manejo das DoMEs^(24,30)^. Por isso, é fundamental que as instituições considerem essas intervenções e avaliem sua eficácia de acordo com o tipo de dor, evitando limitar-se apenas a tratamentos físicos para reduzir a dor ou à concessão de licenças médicas aos profissionais de enfermagem.

O estudo realizado com enfermeiras hospitalares na Espanha^(42)^ incluiu componentes dos três níveis de prevenção durante 12 meses de acompanhamento. Os resultados demonstraram uma redução de 63% no risco de dor no pescoço, ombros e parte superior das costas, mas não apresentaram significância estatística para dor lombar. Por sua vez, estudos mostram que intervenções com abordagem biopsicossocial multidisciplinar são marginalmente mais eficazes para a reabilitação da dor lombar crônica^(53)^.

Outro estudo focou na ergonomia no local de trabalho, abordando o diagnóstico de riscos ergonômicos^(43)^. Embora sua qualidade metodológica e risco de viés limitem a reprodução dos resultados, é importante mencionar que ele evidenciou que a capacitação na identificação e prevenção de riscos musculoesqueléticos e o treinamento aplicado aos riscos durante a mobilização e transferência de pacientes apresentam resultados favoráveis na diminuição da intensidade dos distúrbios musculoesqueléticos, alinhando-se com as recomendações de outras intervenções^(42,68)^ e de organizações do trabalho^(24)^, as quais concordam que a formação ergonômica e o ajuste do local de trabalho podem reduzir o risco ergonômico, melhorar os sintomas musculoesqueléticos e a produtividade. Em contrapartida, não se recomenda combiná-los com capacitação em gestão do estresse laboral, por não ter sido encontrado um efeito significativo^(69)^.

Embora os resultados sejam positivos, é importante considerar que a ergonomia se concentra em adaptar as condições de trabalho e identificar riscos, sendo apenas um indicador de um problema maior no ambiente de trabalho^(20)^.

Outras estratégias, descritas em estudos não incluídos nesta revisão, relatam que ajustar os horários de trabalho pode reduzir o risco de dor nos ombros. Evitar jornadas de trabalho excessivamente longas (mais de 46 horas semanais) pode prevenir dor nos ombros e pescoço^(70)^. A disponibilidade e o uso de equipamentos mecânicos para mobilização e transferência de pacientes estão associados à redução do risco de dor no ombro^(71)^. Revisões sistemáticas^(15,72)^ relataram que o uso de elevadores mecânicos ou deslizantes está relacionado à redução da prevalência de lesões e dor relacionadas ao trabalho entre profissionais de saúde, incluindo os profissionais de enfermagem. Outros estudos^(2,73-74)^ referem que fatores como turnos descontínuos, noturnos e trabalhar em áreas como centros cirúrgicos, serviços de cirurgia, emergência, unidade de terapia intensiva, ginecologia e obstetrícia^(75-78)^ têm sido relacionados a um maior risco de DoMEs. Por isso, os gestores devem considerar essas variáveis ao planejar a distribuição dos serviços e turnos, adaptando-os às necessidades individuais do pessoal com DME.

Nesta investigação, não foram recuperados estudos experimentais que sustentassem o efeito de adequações ergonômicas, assim como de implementações ou mudanças na infraestrutura, melhorias de espaços físicos, mobiliário ergonômico, ou estratégias de dimensionamento de pessoal, organização dos serviços ou dos processos do local de trabalho. Essas intervenções poderiam contribuir para reduzir as DoMEs e a DME, assim como facilitar a reabilitação ou garantir um retorno seguro às atividades laborais^(24,79-80)^. É possível que a falta de evidências sólidas se deva aos custos associados e às políticas de gestão organizacional dos hospitais. No entanto, é relevante considerar essas necessidades para proporcionar uma atenção integral no ambiente laboral do pessoal de enfermagem e a adoção de estratégias de intervenção mais eficazes.

Destaca-se a importância de implementar intervenções efetivas no ambiente laboral hospitalar para prevenir que os profissionais de enfermagem desenvolvam DoMEs, assim como para garantir que aqueles que já apresentam DME possam continuar trabalhando em condições dignas, em um ambiente adaptado às suas condições físicas e psicológicas.

Limitações: o número limitado de estudos experimentais e pré-experimentais sobre o tema, a qualidade da metodologia, o pequeno tamanho amostral, o número de estudos com risco de viés e o número de intervenções implementadas foram fatores limitantes para uma análise mais profunda dos resultados, o que impediu uma metanálise. A especificidade da população de enfermagem na identificação dos estudos pode ter omitido alguns estudos realizados com outros profissionais da saúde, incluindo profissionais de enfermagem.

## Conclusão

Esta revisão mostrou que as estratégias mais efetivas são aquelas que combinam as ações individuais dos profissionais de enfermagem e a melhoria das condições de trabalho, considerando aspectos físicos, psicossociais e ergonômicos para a adaptação do ambiente laboral, prevenir a progressão da dor musculoesquelética e manter o desempenho laboral.

As estratégias mais efetivas para enfrentar a DME nos profissionais de enfermagem hospitalar são o exercício, os alongamentos, a auriculoterapia e o *mindfulness*. Promover essas estratégias para o autocuidado é fundamental, principalmente nos ambientes hospitalares com jornadas prolongadas e nos serviços que geram mais carga física e emocional. As estratégias mais efetivas relacionadas ao ambiente laboral hospitalar são aquelas que adotam uma abordagem integral, combinando aspectos físicos, psicossociais e ergonômicos, direcionados tanto aos funcionários quanto aos empregadores. Isso pode prevenir a progressão dos distúrbios musculoesqueléticos e permite que os profissionais possam continuar desempenhando suas atividades laborais.

Para as perspectivas futuras, é crucial realizar ensaios clínicos randomizados que avaliem de maneira integral estratégias voltadas às necessidades individuais, terapias alternativas e adaptações específicas nos locais de trabalho para beneficiar os profissionais de enfermagem que sofrem de DME.

## Data Availability

Todos os dados gerados ou analisados durante este estudo estão incluídos neste artigo publicado.

# Translation: Estrategias de adaptación de los profesionales de Enfermería con dolor musculoesquelético en el trabajo hospitalario: revisión sistemática

**DOI:** 10.1590/1518-8345.7812.4724

**Authors:** Jorge Gabriel Tuz-Colli, Yolanda Flores-Peña, Heloisa Ehmke Cardoso dos Santos, Fernanda Ludmilla Rossi Rocha, Maria Helena Palucci Marziale

**Affiliations:** 1 Universidad Autónoma de Nuevo León, Facultad de Enfermería de Nuevo León, Monterrey, Nuevo León, México. Universidad Autónoma de Nuevo León Facultad de Enfermería de Nuevo León Nuevo León Monterrey México; 2 Universidade de São Paulo, Escola de Enfermagem de Ribeirão Preto, Centro Colaborador de la OPS/OMS para el Desarrollo de la Investigación en Enfermería, Ribeirão Preto, SP, Brasil. Universidade de São Paulo Escola de Enfermagem de Ribeirão Preto Centro Colaborador de la OPS/OMS para el Desarrollo de la Investigación en Enfermería SP Ribeirão Preto Brasil

**Keywords:** Dolor Musculoesquelético, Habilidades de Afrontamiento, Condiciones de Trabajo, Ergonomía, Enfermeras Practicantes, Enfermería

## Abstract

analizar la efectividad de las estrategias de afrontamiento individual y de adaptación del ambiente laboral hospitalario para profesionales de Enfermería con dolor musculoesquelético.

revisión sistemática de acuerdo a los *Preferred Reporting Items for Systematic reviews and Meta-Analyses*. La búsqueda se realizó en seis bases de datos y se incluyeron estudios de diseño experimental y pre-experimental. El riesgo de sesgo se evaluó mediante las herramientas RoB2 (*Risk of Bias 2)* y ROBINS-I (*Risk of Bias In Non-Randomized Studies of Interventions*) mientras que la calidad metodológica a través del modelo del *Joanna Briggs Institute*, la Escala de Jadad y el Nivel de Evidencia Melnyk, Buck y Gallagher-Ford.

se incluyeron ocho estudios y se identificaron las siguientes estrategias de afrontamiento individual: ejercicios de estiramiento, auriculoterapia y *mindfulness*. Las estrategias aplicadas en el ambiente laboral son multidisciplinarias e integradas y están orientadas a la capacitación en identificar y prevenir riesgos musculoesqueléticos y a mejorar las condiciones del entorno de trabajo.

las estrategias más efectivas son las que combinan las acciones individuales de cada trabajador y la mejora en las condiciones de trabajo considerando aspectos físicos, psicosociales y ergonómicos para adaptar el entorno laboral, prevenir la progresión del dolor musculoesquelético y mantener el desempeño profesional. Registro PROSPERO CRD42024575014.

## Destacados

**(1)** Se han aplicado estrategias individuales y en el lugar de trabajo para reducir el DME. **(2)** Estrategias no farmacológicas como *mindfulness* y auriculoterapia reducen el DME. **(3)** Aplicar la ergonomía en el trabajo reduce el DME. **(4)** Deben considerarse las necesidades individuales de cada trabajador para reducir el DME.

## Introducción

El dolor musculoesquelético (DME) afecta huesos, músculos, ligamentos, tendones y nervios. Es el principal síntoma en los trastornos musculoesqueléticos (TME), se presenta con mayor prevalencia en las regiones de la espalda, del cuello y de los hombros y se asocia a enfermedades como la osteoartritis y la artritis reumatoide, así como al dolor causado por esguinces musculares, fracturas y traumatismos, entre otros^(1-2)^.

Los TME acompañados de DME son un grave problema de Salud Pública que afecta a la población trabajadora, especialmente a los profesionales de la salud y con mayor prevalencia a los de Enfermería. Estos trastornos son la principal causa de ausentismo laboral, limitación de las capacidades funcionales e incapacidad permanente en este personal. Además, suelen provocar limitación de la movilidad y la destreza, lo que reduce la capacidad para trabajar y aumenta el ausentismo y las jubilaciones tempranas, además de incrementar los costos en la atención a la salud, afectar la seguridad de los pacientes y reducir la calidad de vida^(3-5)^.

Se entiende por “profesionales de Enfermería” al conjunto de personas calificadas y autorizadas para ejercer la actividad, incluyendo enfermeros, técnicos, auxiliares y personal de apoyo, conforme a la normativa de cada país. Representan aproximadamente el 59% del total de trabajadores sanitarios, siendo los más numerosos en los hospitales^(6)^. Debido a su alta representatividad, a la diversidad y exigencia de sus funciones y a su constante exposición a riesgos de trabajo (como el manejo de pacientes, el transporte de objetos pesados y la participación en procedimientos clínicos), los profesionales de Enfermería que trabajan en hospitales son vulnerables a desarrollar TME^(7)^.

Un estudio multicéntrico realizado en países de economías emergentes^(8)^ reveló una prevalencia de TME del 37% en la población trabajadora, incrementándose al 92% en los profesionales de Enfermería hospitalaria. Estudios de metaanálisis^(9)^ indican una alta prevalencia del 64% en la región lumbar y del 40% en cuello y hombros^(10)^. Además, el 33% de las bajas laborales en este grupo están vinculadas con los TME^(10)^. El dolor lumbar se destaca como el principal factor que contribuye a la carga general de estos trastornos y es la causa más común de discapacidad en 160 países^(3,11)^.

La prevención de los TME relacionados con el trabajo es muy importante y debe ser objeto de acciones preventivas en el entorno laboral de los profesionales de Enfermería, así como el uso de estrategias de adaptación para que un trabajador que se enferme por TME y padezca DME pueda permanecer activo. Afrontar el dolor es esencial para la calidad de vida y las actividades de la vida diaria; sin embargo, la investigación sobre el tema se ha centrado en estudiar la prevalencia del DME y los factores de riesgo (psicosociales, demográficos, físicos y mentales), además de intervenciones dirigidas a prevenir el problema, cuya implementación en la práctica no ha sido óptima^(12)^ dada la alta prevalencia del DME a nivel mundial, incluso en países con economías de altos ingresos^(13-15)^.

Los pocos estudios sobre estrategias de afrontamiento para el DME en profesionales de Enfermería señalan el uso de diversas estrategias, algunas incluso no recomendables. Entre las más utilizadas se distingue recurrir a analgésicos como Paracetamol y antinflamatorios no esteroideos (AINEs); otras estrategias incluyen ignorar el dolor, buscar consultas médicas con especialistas, optar por medicamentos alternativos, terapias complementarias como masajes terapéuticos, aromaterapia, acupuntura, quiropráctica y *reiki*, autocontrolar el dolor, reposo en cama; fisioterapia y ejercicios para el manejo del dolor^(8,16-17)^.

Investigaciones recientes han distinguido la relación de los factores individuales/psicosociales y la ergonomía con el desarrollo de daños musculoesqueléticos relacionados al trabajo. Este hallazgo contrasta con la consideración tradicional que atribuía estas lesiones principalmente a la exposición a factores de naturaleza biomecánica^(18-20)^.

La ergonomía busca adaptar el entorno laboral a las necesidades de cada trabajador^(21-22)^. En el entorno hospitalario, aplicar principios de ergonomía puede prevenir la aparición y progresión de los TME y del DME, así como mejorar la seguridad y el estado de salud de los profesionales de Enfermería y, en consecuencia, su calidad de vida laboral^(19,20,23)^.

Afrontar el DME es esencial para preservar la productividad de los profesionales de Enfermería, cuyo desempeño no solo representa su sustento económico sino también una fuerza laboral necesaria para atender la creciente demanda de servicios de salud. Esto se agrava ante la escasez global de 5.9 millones de profesionales de Enfermería, principalmente en regiones como África, Sudeste Asiático, Mediterráneo Oriental y países de América Latina como Republica Dominicana, Honduras, Guyana y Venezuela, según lo informado por la Organización Panamericana de la Salud (OPS) en el año 2020^(6)^.

Para aliviar el DME, los profesionales de Enfermería emplean estrategias de afrontamiento tanto individuales como en el lugar de trabajo a fin de mantener su desempeño y prevenir el progreso del problema. Sin embargo, una recuperación completa y reincorporación segura a sus actividades laborales requieren que empleadores y directivos por igual implementen estrategias como dotación de equipos para el traslado de pacientes e implementación de horarios flexibles, adaptando las condiciones laborales a las necesidades y capacidades de cada trabajador (aplicación de la ergonomía). Estas adaptaciones reducen el riesgo de exacerbaciones y accidentes, especialmente en trabajadores con capacidad funcional reducida^(24)^.

Sin embargo, de acuerdo con la revisión de la literatura, se verificó que la información disponible respecto a las estrategias de afrontamiento individual, así como de adaptación en el lugar de trabajo en profesionales de Enfermería, son limitadas. Por lo tanto, se realizó esta revisión sistemática con el objetivo de analizar la efectividad de las estrategias de afrontamiento individual y de adaptación del ambiente laboral hospitalario para profesionales de Enfermería con dolor musculoesquelético.

## Método

Revisión sistemática de la literatura registrada en la plataforma *International Prospective Register of Systematic Reviews* (PROSPERO), Número de registro CRD42024575014 con fecha 16 de agosto de 2024, desarrollada de acuerdo con las recomendaciones de la guía para revisiones del *C*e*ntre for Reviews and Dissemination*(CRD)^(25)^ de los *Preferred Reporting Items for Systematic reviews and Meta-Analyses* (PRISMA)^(26)^ y siguiendo estas etapas: a) Definición de la pregunta de investigación, b) Protocolo de revisión: criterios de exclusión e inclusión, c) Búsqueda bibliográfica: desarrollo de la estrategia de búsqueda, d) Selección de estudios, e) Extracción de datos, f) Evaluación crítica de la calidad de los estudios incluidos, g) Síntesis de los resultados y h) Presentación y difusión de los resultados.

### Definición de la pregunta de investigación

La pregunta de investigación fue la siguiente: “¿Qué tan efectivas son las estrategias de afrontamiento individual y de adaptación del entorno laboral hospitalario para los profesionales de Enfermería con dolor musculoesquelético?”. La pregunta se estructuró sobre la base de la estrategia PICOS^(27)^ (acrónimo de *Population*, *Interventions*, *Comparison*, *Outcome*, *Study design*), donde P (Participantes): profesionales de Enfermería que trabajan en hospitales (enfermeros, técnicos y auxiliares de enfermería) y sufren DME; I (Intervención): estrategias de afrontamiento individual y en el ambiente de trabajo para la adaptación de profesionales de Enfermería con DME; C (Comparación/Control): no correspondió su aplicación; y O (*Outcome*/Resultado): adaptación de los profesionales de Enfermería en el ambiente de trabajo.

Definiciones: a) Profesionales de Enfermería: enfermeros, técnicos de enfermería, auxiliares de enfermería. b) DME: experiencia sensorial y emocional desagradable que afecta huesos, músculos, ligamentos, tendones y nervios; es el principal síntoma en los trastornos musculoesqueléticos.

### Protocolo de revisión: criterios de inclusión y exclusión

Criterios de inclusión: estudios primarios que respondieran la pregunta de investigación sobre la efectividad de las estrategias de afrontamiento individual y de adaptación aplicadas en el ambiente laboral para profesionales de Enfermería con DME. Se incluyeron estudios de diseño experimental (ensayos clínicos aleatorizados - ECA, ensayos clínicos - EC) y estudios pre-experimentales (pre- y pos-test con un grupo), así como artículos sin límite de idioma y publicados entre enero de 2000 y julio de 2024.

Criterios de exclusión: estudios que no respondieran la pregunta de investigación, profesionales de Enfermería que no trabajaran en hospitales, estudios piloto, descriptivos y observacionales y revisiones de literatura de todo tipo.

### Búsqueda bibliográfica: desarrollo de la estrategia de búsqueda

Las búsquedas se realizaron en las siguientes bases de datos: MEDLINE/*PubMed*^®^, *Web of Science*, *Scopus*, *Cochrane Library*, *Cumulative Index to Nursing and Allied Health Literature* (CINAHL*)* y Literatura Latinoamericana y del Caribe en Ciencias de la Salud (LILACS). La estrategia de búsqueda se implementó mediante una combinación de descriptores controlados y palabras clave, utilizando los operadores booleanos “*OR*” entre sinónimos y similares y “*AND*” entre palabras clave, conforme a las particularidades de cada base de datos. El desarrollo de la búsqueda fue asesorado técnicamente por una bibliotecóloga. La estrategia se estructuró utilizando el vocabulario controlado de los *Medical Subject Headings* (MeSH) y de los Descriptores en Ciencias de la Salud (DeCS). La búsqueda en las bases de datos se hizo entre el 31 agosto y el 7 septiembre del 2024, incluyendo artículos publicados entre enero de 2000 y julio de 2024. Las referencias se administraron en el programa de *softwareMendeley*. En la Figura 1 se ilustra la estrategia de búsqueda.

**Figura 1-:** Estrategias de búsqueda. Ribeirão Preto, SP, Brasil, 2024

*PubMed* /MEDLINE (“Nursing Assistant*” OR “Personnel nursing” OR Nurse* OR “nursing worker*” OR “nursing professional*”) AND (“Musculoskeletal Pain”[Mesh] OR “Musculoskeletal Pain” OR “muscle pain”)
*Web of Science* (“Nursing Assistant*” OR “Personnel nursing” OR Nurse* OR “nursing worker*” OR “nursing professional*”) AND (“Musculoskeletal Pain” OR “muscle pain”)
*Scopus* (“Nursing Assistant*” OR “Personnel nursing” OR Nurse* OR “nursing worker*” OR “nursing professional*”) AND (“Musculoskeletal Pain” OR “muscle pain”)
*Cochrane* ((MeSH descriptor: [Nursing NEXT Assistant*] explode all trees) OR (Personnel NEXT nursing) OR Nurse OR Nurses OR (nursing NEXT worker*) OR (nursing NEXT professional*)) AND ((MeSH descriptor: [Musculoskeletal Pain] explode all trees) OR “Musculoskeletal Pain” OR “muscle pain”)
CINAHL (“Nursing Assistant*” OR “Personnel nursing” OR Nurse* OR “nursing worker*” OR “nursing professional*”) AND ((MH “Musculoskeletal Pain”) OR “Musculoskeletal Pain” OR “muscle pain”)
BVS – LILACS (“Profissional de enfermagem” OR “Profissionais de enfermagem” OR “Profissionais da enfermagem” OR “Técnico de enfermagem” OR “trabalhadores da enfermagem” OR “trabalhador da enfermagem” OR “trabalhador de enfermagem” OR “trabajadores de enfermería” OR “trabajador de enfermería” OR “Assistentes de Enfermagem” OR “Auxiliar de Enfermagem” OR “Auxiliar do Enfermeiro” OR “Auxiliares de Enfermagem” OR “Auxiliares de Enfermeiros” OR “Asistentes de Enfermería” OR “asistente de enfermería” OR “auxiliar de enfermería” OR “auxiliar del enfermero” OR “auxiliares de enfermeros” OR “auxiliares de enfermería” OR enfermeri* OR Enfermero* OR Enfermera* OR “Healthcare Assistant*” OR “Nursing Assistant*” OR “Personnel nursing” OR “nursing professional* OR Nurse* OR”nursing worker*” OR “nursing professional*” OR Enfermagem OR Enfermería OR Nursing) AND (“Dor Musculoesquelética*” OR “Dores Musculoesqueléticas*” OR “Dor muscular*” OR “Dores musculares” OR “Dor Osteomuscular*” OR “dolor osteomuscular*” OR “Dolor Musculoesquelético” OR “Musculoskeletal Pain” OR “muscle pain”)

### Selección de estudios

Los resultados de la búsqueda en las bases de datos se exportaron a la plataforma de *software* en línea *Rayyan®*^(28)^, utilizada para almacenar, organizar, eliminar duplicados y seleccionar a ciegas los estudios. Los materiales fueron seleccionados en dos fases. La primera mediante el análisis de los títulos y resúmenes, realizado por dos revisores de forma independiente y anónima, siguiendo los pasos del diagrama de flujo PRISMA^(26)^. En la segunda fase se analizaron los artículos a texto completo seleccionados y se aplicaron los criterios de inclusión, de forma enmascarada (revisores R1 y R2) con la finalidad de limitar el sesgo en la revisión (septiembre de 2024). La resolución de conflictos por discrepancias entre los estudios estuvo a cargo de otro investigador experto en el tema (R3) a través de la discusión de los criterios de selección.

Los revisores emplearon las siguientes definiciones para guiar las decisiones:

Afrontamiento individual de trabajadores con DME: son las acciones cognitivas y conductuales dinámicas que los profesionales de Enfermería desarrollan para enfrentar situaciones estresantes y aliviar/afrontar el DME para mantener sus actividades en el lugar de trabajo^(29)^.

Adaptación del ambiente de trabajo para trabajadores con DME: alcanzada mediante intervenciones en el lugar de trabajo (ambiente laboral) como actividades, tareas y productos desarrollados o implementados para cambiar o reducir la acción de los riesgos laborales e incrementar los conocimientos, los comportamientos y las actitudes de los trabajadores. Estas intervenciones pueden desarrollarse directamente en el ambiente de trabajo o estar dirigidas a los trabajadores a través de educación y formación continua y adecuando el ambiente laboral a las características psicofisiológicas de los profesionales de Enfermería^(30)^.

### Extracción de datos

Para extraer los datos de los estudios seleccionados se empleó una tabla de caracterización elaborada en *Microsoft Excel*^®^ que incluyó lo siguiente: año de publicación, país de origen, objetivo, tipo de diseño del estudio, criterios de inclusión y exclusión de la muestra, detalles del tipo y método de análisis de los datos, descripción de la intervención, instrumento de evaluación, resultados, conclusiones y nivel de evidencia.

Se extrajeron los hallazgos de mayor relevancia y las conclusiones de los estudios seleccionados, relacionados con la pregunta de revisión, y se los analizó a partir de dos categorías previas, a saber:

Intervenciones y/o estrategias de afrontamiento que realizan los profesionales de Enfermería que sufren DME relacionado con el trabajo.

Intervenciones y/o estrategias para promover la adaptación de los profesionales de Enfermería con DME implementadas y/o adoptadas en el ambiente de trabajo.

### Evaluación crítica de la calidad de los estudios incluidos

Se utilizaron diversas herramientas para evaluar la calidad metodológica de los estudios susceptibles de revisión, considerando tanto el método utilizado como el contenido; para ello, aplicamos las herramientas de evaluación crítica del *Joanna Briggs Institute* (JBI)^(31)^. Para el análisis de la calidad metodológica de los ensayos clínicos aleatorizados (ECA) se utilizó la escala de Jadad^(32)^, que evalúa tres ítems de un estudio a través de cinco preguntas binarias con respuestas del tipo “Sí” (vale 1) o “No” (vale 0). Los ítems analizan la aleatorización, el enmascaramiento, las pérdidas y los abandonos de participantes para obtener una puntuación general de 0 a 5. Los resultados de 0 a 2 se consideran de “baja calidad metodológica” y los de 3 a 5 de “alta calidad metodológica”.

Para determinar el nivel de evidencia de los estudios seleccionados seguimos el marco establecido por Melnyk, Buck y Gallagher-Ford^(33)^. Este paso también estuvo a cargo de revisores que trabajaron de forma independiente y enmascarada. Se convocó a un tercer revisor para resolver posibles conflictos en la evaluación.

### Evaluación del riesgo de sesgo

El riesgo de sesgo de los ensayos clínicos aleatorizados fue evaluado por dos revisores independientes mediante la herramienta *Risk of Bias 2* (RoB2)^(34)^. Para los estudios clínicos no aleatorizados pre-experimentales se empleó la herramienta *Risk of Bias In Non-Randomized Studies of Interventions* (ROBINS-I)^(35)^.

### Aspectos éticos

Este estudio secundario no requirió aprobación de un Comité de Ética en Investigación. Sin embargo, los autores declaran que no existen conflictos de intereses que comprometan el análisis de los resultados de este trabajo.

## Resultados

Se recuperó un total de 1732 estudios: 316 (18,2%) artículos en *PubMed*/Medline; 435 (25,1 %) en *Scopus*; 417 (24,1%) en *Web of Science*; 281 (16,2%) en *CochraneLibrary*; 213 (12,3%) en CINAHL; y 70 (4%) en LILACS. Tras eliminar registros duplicados (n=711), se identificaron 1021 estudios para revisar sus títulos y resúmenes, dejando 33 publicaciones potenciales para una revisión de los artículos completos y, después de dicha revisión, se incluyeron 8 artículos científicos en este estudio.

En la Figura 2 se muestra el diagrama de flujo de la búsqueda en la literatura y de los criterios de selección adaptado de PRISMA^(26)^.

**Figura 2-:** Diagrama de flujo de la búsqueda en la literatura y de los criterios de selección adaptado de PRISMA^(26)^

Con respecto a las características de los estudios, todos los artículos estaban publicados en inglés; se publicaron 3 (37,5%) estudios en Brasil, 2 (25,0%) en Japón, 1 (12,5%) en España, 1 (12,5%) en Finlandia y 1 (12,5%) en Turquía. Respecto a la población estudiada, en 4 (50,0%) estudios participaron enfermeras y en otros 4 (50,0%) trabajadores de la salud que incluyeron principalmente enfermeras.

### Síntesis de la evidencia

El análisis del nivel de evidencia de los estudios se realizó a través del marco establecido por Melnyk, Buck y Gallagher-Ford. Un total de 8 estudios experimentales clasificaron en el Nivel II, a saber: 7 ECA^(36-42)^ y 1 estudio pre-experimental^(43)^.

En la evaluación de la calidad metodológica de los 7 ECA sobre la base de la escala de Jadad^(32)^, se descubrió que 6 (85,7%) estudios fueron de alta calidad metodológica^(36-39,41-42)^ y uno (14,30%) de baja calidad^(40)^. El estudio pre-experimental^(43)^ calificó como de baja calidad metodológica.

Los instrumentos de evaluación del DME variaron según cada estudio; incluyeron el uso de la Escala Visual Análoga (EVA), adaptaciones del cuestionario Nórdico de síntomas musculoesqueléticos y el Cuestionario de creencias sobre el miedo y la evitación (*Fear-Avoidance Beliefs Questionnaire* FABQ).

Los estudios analizados evaluaron la efectividad de diversas intervenciones implementadas en contextos hospitalarios, dirigidas a profesionales de Enfermería con DME asociado al trabajo. Las estrategias de afrontamiento individual reportadas incluyeron las siguientes: ejercicios terapéuticos, Pilates, estiramientos activos y progresivos^(36-39)^, terapias como *mindfulness* adaptado^(40)^, auriculoterapia^(41)^ y educación en neurociencia del dolor^(39)^. Las intervenciones dirigidas a favorecer la adaptación en el ambiente de trabajo comprendieron una intervención multifacética con enfoque participativo en los tres niveles de prevención^(42)^ y un programa de gestión de riesgos ergonómicos^(43)^.

En la Figura 3 se presentan los resultados de la evaluación del riesgo de sesgo en los estudios.

En las Figuras 4 y 5 se presentan las características de los estudios incluidos en la revisión.

**Figura 3-:** Evaluación del riesgo de sesgo en los estudios incluidos por medio de las herramientas RoB2 (*Risk of Bias 2)* y ROBINS-I (*Risk of Bias In Non-Randomized Studies of Interventions.* Ribeirão Preto, SP, Brasil, 2024

**Figura 4–:** Características de los estudios incluidos en la revisión sobre las estrategias o acciones de afrontamiento que realiza el personal de Enfermería con DME en relación con el trabajo (n = 8). Ribeirão Preto, SP, Brasil, 2024

Primer autor, año, país	Diseño del estudio	Nivel de evidencia/ Calidad metodológica	Participantes	Intervención	Resultados
Moreira, et al., 2021 ^( 36 )^ ; Brasil	Experimental, ensayo clínico controlado aleatorizado, simple ciego, de grupos paralelos con ocultación de la asignación	Nivel II 3	GI*: 46 auxiliares de enfermería; y 44 en el GC ^†^	El programa se aplicó dos veces por semana con 30 minutos por sesión. El entrenamiento estuvo compuesto por ejercicios para estabilizar la columna. Cada sesión constó de tres pasos: 1) 5 minutos de ejercicios de calentamiento; 2) 20 minutos de ejercicios terapéuticos; 3) 5 minutos de estiramiento de los músculos isquiotibiales y ejercicios de enfriamiento al final de la sesión.	Los participantes mostraron una mejora en la fuerza de los músculos flexores del tronco con tamaño de efecto de 0,77 ( *p* =0,002) y en el control de los síntomas de la espalda baja con 6,25 ( *p* =0,002) después del programa de ejercicios terapéuticos. La asociación positiva entre la exposición al ejercicio y la mejoría de los síntomas fue 2,04 veces mayor para el GI* que en el GC ^†^ (OR ^‡^ =0,04; IC ^§^ 95%: 1,82-2,72).
Taulaniemi, et al., 2020 ^( 37 )^ ; Finlandia	Experimental, ensayo clínico controlado aleatorizado de cuatro ramas	Nivel II 3	GI*: 165 trabajadoras de la salud; 54 GC ^†^ El 87% eran enfermeras	Programa de ejercicios tipo Pilates, centrado en el control de la postura neutra de la columna dividido en tres etapas. Etapa I. Clases de ejercicios neuromusculares supervisados. Etapas II y III: una clase supervisada y una sesión en casa con ayuda de un DVD o un folleto elaborado para el estudio. En esta etapa se les permitió hacer ejercicio en sesiones grupales supervisadas.	La práctica de Pilates con buena adherencia redujo los niveles de creencias sobre el miedo y la evitación (sigla en inglés: FAB) relacionado con el trabajo con la reducción de la intensidad del dolor lumbar ( *r* =0,16; *p* =0,05). Se identificó que las estrategias de motivación deben enfocarse en personas con niveles bajos de educación, menor condición física y altos niveles de FAB para lograr una mejor adherencia al ejercicio.
Oka, et al., 2019 ^( 38 )^ ; Japón	Analítico, prospectivo, aleatorizado, de grupos paralelos y multicéntrico con evaluaciones centrales	Nivel II 3	4767 enfermeras Grupo A: 1799; Grupo B; 1420; y 1548 en el Grupo C, de 12 hospitales	El grupo A fue el grupo control. Los participantes de los grupos B y C recibieron un manual de ejercicios que describía cómo hacer el ejercicio de extensión de espalda de pie “ *One Stretch* ”, y un seminario de 30 minutos. Los participantes del grupo B solo recibieron el seminario de 30 minutos. En el grupo C, utilizaron enfoques físicos y psicológicos para el tratamiento del dolor lumbar.	El ejercicio de extensión de espalda de pie “ *One Stretch* ” mejoró y previno eficazmente el dolor lumbar. Las tasas de mejoría en los grupos A, B y C fueron del 13,3%, 23,5% y 22,6%, respectivamente. Las tasas de empeoramiento del dolor fueron del 13,0%, 9,6% y 8,1%, que disminuyeron a medida que aumentó el grado de intervención ( *p* <0,001).
Imai, et al., 2021 ^( 39 )^ ; Japón	Experimental, ensayo controlado aleatorizado de grupos paralelos	Nivel II 3	104 participantes 53 en el GI* y 51 en el GC ^†^ y trabajadores de salud incluidos enfermeros	Consistió en un plan de seis meses de ejercicios progresivos y educación en neurociencia del dolor (END). Con estiramientos de 20 minutos y caminatas de aproximadamente 30 minutos, 3-4 veces por semana. El grupo control recibió retroalimentación general después de responder un cuestionario.	El estudio demostró que una combinación de END y ejercicio dio mejores resultados para los pacientes, menor presentismo *F=* 12,87; *p* =<0,001; η ^2^ =0,94), menor intensidad del dolor *F* =11,0; *p* =<0,001; η ^2^ =0,1), menor estrés físico y mejor estado psicológico y calidad de vida.
Lopes, et al., 2019 ^( 40 )^ ; Brasil	Experimental, ensayo clínico, prospectivo y abierto de medidas repetidas	Nivel II 2	GI*: 64 técnicos de enfermería Sin GC ^†^	Programa de *mindfulness* adaptado con sesiones semanales de 60 minutos, durante 8 semanas consecutivas. Los participantes fueron guiados para practicar meditación en casa diariamente durante 20 minutos, incluso cuando realizaban sus rutinas y actividades diarias como una práctica informal. En las reuniones cara a cara, los ejercicios se centraron en manejo del dolor, técnicas de respiración, escaneo corporal, caminata consciente, movimientos conscientes con posturas corporales ligeras, sentarse, acostarse y meditación.	Los resultados del programa de *mindfulness* indicaron una reducción significativa en los síntomas musculoesqueléticos y en los niveles de ansiedad, depresión y dolor catastrófico ( *p* <0,001). Un hallazgo muy significativo fue la disminución en los niveles de dolor catastrófico (η ^2^ =0,203; *p* <0,001) y DME (η ^2^ =0,200; *p* <0,001). Los efectos positivos se identificaron después de 8 semanas y se mantuvieron sin cambios hasta la semana 12 de seguimiento.
Morais, et al., 2023 ^( 41 )^ ; Brasil	Experimental, ensayo clínico aleatorizado, triple ciego (paciente, estadístico y evaluadores de resultados)	Nivel II 5	GI*: 34 trabajadores; 33 en el GC ^†^ , trabajadores de la salud (incluido personal de Enfermería) con diagnóstico de dolor raquídeo crónico	Se ofrecieron ocho sesiones de auriculoterapia| con semillas en dos aplicaciones por semana, con una duración promedio de 10 a 15 minutos. Se instruyó a los trabajadores dejar por 3 días con estimulación diaria de forma manual al menos tres veces al día, 15 veces en cada punto auricular.	La auriculoterapia con semillas presentó efectos positivos como práctica terapéutica en reducir la intensidad del dolor entre los trabajadores de la salud que presentaban dolor crónico en la columna vertebral con una reducción significativa del 34% en el GI* con relación al GC ^†^ ( *p* =0,007). En cuanto a la interferencia del dolor en las actividades de la vida diaria se observó una reducción de los valores medios en ambos grupos ( *p* <0,05). También se notó una reducción en el uso de medicamentos ( *p* =0,013).
Soler-Font, et al., 2019 ^( 42 )^ ; España	Experimental, ensayo clínico controlado aleatorizado por conglomerados de dos ramas con grupo control	Nivel II 3	GI*: 138 enfermeras; y 119 en el GC ^†^	Se emplearon componentes en los tres niveles de prevención. Los componentes de prevención primaria fueron: 1) factores de riesgo ocupacional para proteger a los trabajadores sanos de los TME y las ausencias por enfermedad relacionadas con los TME a través de la ergonomía participativa; y 2) promoción de estilos de vida saludables en el trabajo (actividad física, bienestar emocional y dieta). La prevención secundaria y terciaria comprendía un servicio de gestión de casos para identificar DME temprano.	La intervención fue efectiva para reducir el dolor hasta un 63% (OR ^‡^ =0,37; IC ^§^ del 95%: 0,14-0,96) en cuello, hombros y espalda superior en el personal de Enfermería. Se mostró una reducción no estadísticamente significativa del dolor lumbar en el grupo intervención en comparación con los grupos control. El funcionamiento laboral se mantuvo estable a lo largo de los 12 meses de seguimiento en el grupo intervención en comparación con el grupo control ( *p* <0,05).
Sezgin & Esin, 2018 ^( 43 )^ ; Turquía	Pre-experimental Pre- y post-test para grupos control no equivalentes	Nivel II 2	61 enfermeras; 30 en el GI* y 31 en el GC ^†^	El programa de gestión de riesgos ergonómicos (PGRE) consistió en dos intervenciones: 1) una capacitación en video de dos semanas centrada en los riesgos musculoesqueléticos de las enfermeras de la UCI complementado con ejercicios de prevención; 2) entrevistas personales con las enfermeras enfocadas en identificar los factores predisponentes, reforzadores y facilitadores del cambio de conducta.	El PGRE fue eficaz para aumentar la frecuencia del ejercicio ( *p* =0,017) y disminuir el dolor musculoesquelético ( *p* =0,017) y los niveles de riesgo ergonómico entre las enfermeras de la UCI ( *p* <0,05).

*GI = Grupo intervención; ^†^GC = Grupo control; ^‡^OR = Razón de Probabilidades; ^§^IC = Intervalo de confianza

**Figura 5-:** Descripción de las intervenciones aplicadas en los estudios incluidos (n = 8). Ribeirão Preto, SP, Brasil, 2024

Primer autor, año, país	Intervención/Técnica usada	Cantidad de sesiones	Duración por sesión	Instrumento/Técnica de evaluación	Resultado
Moreira, et al., 2021 ^( 36 )^ ; Brasil	Ejercicio terapéutico para la fuerza muscular de la columna (calentamiento, fortalecimiento y estiramiento)	17,5 sesiones en promedio	30 minutos	Dinamometría: Fuerza de músculos flexores del tronco; umbral de dolor a la presión de dorsal largo	Mayor fuerza en músculos flexores Disminución significativa del umbral de dolor por presión
Taulaniemi, et al., 2020 ^( 37 )^ ; Finlandia	Ejercicio físico tipo Pilates Ejercicio neuromuscular	2 veces por semana los primeros 2 meses. Posteriormente, 1 vez por semana, cono 4 meses de seguimiento	60 minutos	-Cuestionario de creencias sobre el miedo y la evitación (FABQ). -Registro de adherencia al ejercicio	Menor creencia sobre evitación del dolor relacionada con la actividad física y el trabajo Mayor adherencia al ejercicio
Oka, et al., 2019 ^( 38 )^ ; Japón	Ejercicios de extensión activa de la espalda “ *One Stretch* ” Seminario educativo Enfoques psicológicos y físicos	1 seminario y seguimiento de 6 meses	30 minutos	-Herramienta de detección de dolor lumbar *Keele STarT Back* (SBST) -Cuestionario de creencias sobre el miedo y la evitación (FABQ)	Mejoría subjetiva del dolor Disminución del dolor
Imai, et al., 2021 ^( 39 )^ ; Japón	Ejercicios físico progresivo y educación en neurociencia del dolor (END).	3 a 4 veces por semana por 6 meses	Estiramientos de 20 minutos y caminatas de 30 minutos	-Índice de dolor generalizado ( *Widespread Pain Index* , WPI) -EuroQol de 5 dimensiones (EQ5D-L) -Escala de presentismo (WLQ)	Menor dolor lumbar Menor presentismo
Lopes, et al., 2019 ^( 40 )^ ; Brasil	*Mindfulness* adaptado	8 sesiones	60 minutos	-Cuestionario Nórdico de síntomas Musculoesqueléticos -Escala de dolor catastrófico -Escala de Calidad de vida de la OMS (WHOQOL-BREF)	Menor dolor, ansiedad y catastrofismo Mayor percepción de calidad de vida.
Morais, et al., 2023 ^( 41 )^ ; Brasil	Auriculoterapia con semillas	8 sesiones	10-15 minutos	-Escala visual análoga del dolor -Inventario Breve del dolor -Cuestionario de discapacidad de Rolland-Morris y de Calidad de vida SF-36	Menor intensidad del dolor de crónico espinal Mejora en la percepción de calidad de vida
Soler-Font, et al., 2019 ^( 42 )^ ; España	Intervención multifacética en los tres niveles de prevención I. Ergonomía participativa: reducción de exposición a factores de riesgo biomecánico y psicosociales II. Promoción de la salud: caminata nórdica, Alimentación saludable mediterránea y Curso de *Mindfulness* III. Gestión de casos: detección temprana de TME y DME; Apoyo para el regreso al trabajo multidisciplinario	Variable, durante 12 meses	Variable dependiendo del componente	-Cuestionario Nórdico Estandarizado adaptado -Funcionamiento laboral mediante el *Work Role Functioning Questionnaire* , versión en español (WRFQ-SpV)	Menor de dolor en cuello, hombros y espalda alta Funcionamiento laboral estable Menor solicitud de reprogramación del trabajo
Sezgin & Esin, 2018 ^( 43 )^ ; Turquía	Programa de gestión de riesgos ergonómicos basado en el modelo PRECEDE-PROCEDE 1) Capacitación en video y material educativo (folletos y CD) complementado con ejercicios de prevención 2) Entrevistas personales enfocadas en identificar los factores predisponentes, reforzadores y facilitadores del cambio de conducta	26 semanas de seguimiento	2 semanas	-Cuestionario de síntomas musculoesqueléticos -Formulario de informe de riesgos ergonómicos -Formulario de evaluación rápida de extremidades superiores ( *Rapid Upper Limb Assessment* , RULA)	Menor DME Aumento de la frecuencia de ejercicio

## Discusión

El objetivo del estudio fue analizar la efectividad de las estrategias de afrontamiento individual y de adaptación del ambiente laboral hospitalario para profesionales de Enfermería con DME. Aunque este problema está ampliamente reconocido en el ámbito de la profesión^(9-10,15)^, la evidencia científica sólida disponible es limitada. La investigación disponible se ha enfocado en la prevalencia, los factores de riesgo, la causalidad y las intervenciones preventivas relacionadas con los TME y el DME^(12)^. Sin embargo, considerando que vivir sin dolor es un derecho universal^(44)^, resulta crucial encontrar mejores formas de adaptación o alivio del sufrimiento físico de los profesionales de Enfermería, aun cuando el dolor no represente una amenaza inmediata para la vida^(45)^. En este contexto, es necesario adoptar estrategias efectivas de afrontamiento del DME, junto con intervenciones y adaptaciones en el ambiente de trabajo que respondan a sus necesidades. Los estudios analizados en esta revisión aportan evidencia sobre la efectividad de las intervenciones con potencial para prevenir la progresión del DME en los profesionales de Enfermería en entornos hospitalarios. A pesar de la heterogeneidad observada en las estrategias, la cantidad y la duración de las sesiones (que variaron desde intervenciones de 8 sesiones por semana hasta un programa de un año^(42)^), los resultados demostraron cierta disminución en la intensidad del dolor, mejoras en la funcionalidad física y en el desempeño laboral y un aumento en los indicadores de calidad de vida de los profesionales de Enfermería.

Los resultados se presentan de acuerdo con las categorías de análisis establecidas para este estudio.

### Estrategias o acciones de afrontamiento que implementan los profesionales de Enfermería que sufren DME relacionado con el trabajo

Se destaca que los estudios incluidos no detallan las estrategias específicas utilizadas por los profesionales de Enfermería en su práctica profesional, pero que si han demostrado efectividad para afrontar el DME. Estas estrategias pueden ser opciones viables para que las apliquen los profesionales. Las estrategias individuales/personales adoptadas por los profesionales de Enfermería evidenciadas en los ECA presentaron alta calidad metodológica en la mayoría de los estudios^(36-39,42)^, incluido el de auriculoterapia con semillas^(41)^, el cual fue clasificado con cinco puntos en la clasificación de Jadad. Sin embargo, un estudio de un programa de gestión de riesgos ergonómicos mostró baja calidad, con dos puntos^(43)^. Esto indica cierta falta de homogeneidad metodológica en los estudios.

La mayoría de los estudios incluidos se centró en reducir el dolor lumbar debido tanto a su relevancia epidemiológica^(3)^ como a la alta prevalencia entre profesionales de Enfermería, que oscila entre el 44,4% y el 90,2%^(46-49)^. Esta alta prevalencia afecta la productividad laboral y las actividades diarias^(50)^. Las estrategias más efectivas incluyeron ejercicios terapéuticos enfocados en mejorar la estabilización de la columna^(36-37)^ y estiramientos combinados con otras acciones^(38-39,43)^. Estos hallazgos se condicen con los resultados de otras revisiones sistemáticas, según lo reportado por investigadores belgas^(51)^ que evaluaron la eficacia de diversas intervenciones para prevenir y tratar el dolor lumbar en enfermeras; como así también la investigación realizada en Italia, que analizó intervenciones orientadas a prevenir y reducir los TME en profesionales de salud, incluidos los de Enfermería^(15)^.

Desde una perspectiva fisiológica, los ejercicios terapéuticos abordan la debilidad muscular y la movilidad articular limitada, factores que aumentan el riesgo de síntomas musculoesqueléticos^(52)^. Además, producen carga física en el sistema musculoesquelético, lo que aumenta la probabilidad de lesiones por sobrecarga. También se ha demostrado que autoevaluaciones de baja capacidad física predicen el dolor lumbar en trabajadoras de la salud^(52)^. En consecuencia, es importante promover la actividad física y el ejercicio como estrategias de prevención y autocuidado terapéutico en los profesionales de Enfermería con DME.

Las intervenciones que combinaron el ejercicio con otras acciones demostraron ser efectivas en reducir los niveles de DME. Entre ellas, se identificó el uso de enfoques tanto físicos como psicológicos^(36-39,42-43)^. Estas intervenciones combinaron ejercicio físico con componentes psicosociales que también mostraron una reducción autoinformada del miedo al dolor, la ansiedad y el estrés^(38-39)^. La evidencia respalda la efectividad de estas intervenciones, destacando aquellas que incorporan aspectos psicosociales, más efectivas que las de atención habitual o de tratamientos físicos^(53)^. Este hallazgo debe ser considerado tanto por los profesionales de Enfermería como por sus empleadores, no solo como terapia individual sino también para prevenir el DME. El estrés psicológico caracterizado por alta demanda, bajo control y escaso apoyo social se ha asociado con la presencia de DME (OR=1,81; IC 95%: 1,50-2,18)^(18)^. Además, estudios como el realizado en Mumbai-India han reportado una relación similar entre el estrés laboral en profesionales de Enfermería y otras morbilidades musculoesqueléticas^(54)^. Cabe señalar que los factores psicosociales en el entorno laboral pueden actuar como estresantes originando reacciones fisiológicas por estrés, incluyendo procesos bioquímicos que generan tensión muscular a corto plazo y aumentan el riesgo de DME a largo plazo^(53)^. La forma en la que estos factores adversos afectan a cada persona puede variar dependiendo de sus características individuales, los recursos disponibles y las estrategias de afrontamiento utilizadas. Por lo tanto, en el caso de los profesionales de Enfermería es fundamental abordar tanto el estrés relacionado con el trabajo como el estrés personal, ya que ambos son factores de riesgo en el control del DME^(55)^.

El programa terapéutico basado en *Mindfulness*^(40)^ adaptado del *Mindfulness-Based Stress Reduction* (MBSR)^(56)^ redujo significativamente el dolor y también los síntomas musculoesqueléticos, los niveles de ansiedad y la depresión (*p*<0,001). Diversos estudios respaldan su efectividad y seguridad para disminuir la intensidad del dolor lumbar crónico en adultos, incluyendo una reducción en el uso de analgésicos, como también se encuentra en otros estudios de revisión de la literatura^(57-58)^. A pesar de los resultados, su aplicación en la práctica sigue siendo poco conocida.

La auriculoterapia con semillas^(41)^ presentó efectos positivos en reducir la intensidad del dolor entre trabajadores de la salud con dolor crónico. No se encontraron otros estudios similares durante la revisión. Es una terapia poco explorada en el continente americano. Un estudio realizado en Corea^(59)^ reveló que la auriculoterapia disminuyó significativamente el DME en adultos. Además, cuanto más prolongado el período de intervención, mayor fue el tamaño del efecto observado. Esta terapia también ha demostrado resultados positivos en la atención de la salud mental, aliviando el estrés, la ansiedad y la depresión^(60)^. Asimismo, un estudio realizado en unidades perioperatorias demostró los beneficios de la auriculoterapia en el alivio de la ansiedad y el estrés entre profesionales de Enfermería^(61)^. El *Mindfulness* y la auriculoterapia con semillas deben ser opciones para el autocontrol del DME en el personal de Enfermería y se debe fomentar su uso en entornos clínicos.

Cabe señalar que en la revisión se identificaron estudios que no cumplían con los criterios de inclusión; sin embargo, referían acciones que realizan los profesionales de Enfermería para aliviar el DME y merecen ser destacadas, tales como las siguientes: uso de analgésicos^(16,62)^; automedicación (uso de medicamentos alopáticos y homeopáticos), que se ha documentado tiene una prevalencia que oscila entre 30% y el 85,6%^(8,17,48,63-65)^; descanso y fisioterapia^(62)^; postura correcta en el cuidado del paciente; mantener mayor atención en todas las actividades a realizar; realizar funciones con mayor calma y tiempo; y participar en las capacitaciones que ofrecen las instituciones^(66)^; además, otras terapias complementarias que se han reportado fueron aromaterapia, acupuntura, quiropráctica, *reiki* y acupresión^(17,67)^. Las terapias alternativas que se han probado o aplicado en otras poblaciones deben ser consideradas y susceptibles a comprobación por estudios de mayor calidad entre profesionales de Enfermería.

### Intervenciones y/o estrategias para promover la adaptación de los profesionales de Enfermería con DME implementadas y/o adoptadas en el ambiente de trabajo

La evidencia científica destaca que las intervenciones multidimensionales que abarcan los tres niveles de prevención (primario, secundario y terciario) son más efectivas debido a la naturaleza multifactorial del DME relacionado con el trabajo^(51)^. Estas intervenciones coinciden con las directrices de organismos de salud laboral, que aconsejan implementar estrategias preventivas multidisciplinarias e integradas para abordar los TME^(24,30)^. Por ese motivo, es fundamental que las instituciones consideren estas intervenciones y evalúen su efectividad según el tipo de dolor, evitando limitarse únicamente a tratamientos físicos para disminuir el dolor o a otorgar licencias por incapacidad a los profesionales de Enfermería.

El estudio realizado con enfermeras hospitalarias en España^(42)^ incluyó componentes de los tres niveles de prevención durante 12 meses de seguimiento; los resultados demostraron una reducción del 63% en el riesgo de dolor en cuello, hombros y parte superior de la espalda, pero no mostró significancia estadística en el caso del dolor lumbar. A su vez, otros estudios muestran que las intervenciones con enfoque biopsicosocial multidisciplinario son marginalmente más efectivas para la rehabilitación del dolor lumbar crónico^(53)^.

Otro estudio se centró en la ergonomía en el lugar de trabajo, abordando el diagnóstico de riesgos ergonómicos^(43)^. Aunque su calidad metodológica y riesgo de sesgo limitan la reproducción de sus resultados, es importante señalar que evidenció que la capacitación en identificar y prevenir riesgos musculoesqueléticos y la capacitación aplicada en los riesgos durante la movilización y el traslado de pacientes tienen resultados favorables en cuanto a disminuir la intensidad del DME, alineándose con las recomendaciones de otras intervenciones^(42,68)^ y de organismos del trabajo^(24)^, que concuerdan en que la formación ergonómica y el ajuste de los puestos de trabajo pueden reducir el riesgo ergonómico, además de mejorar los síntomas musculoesqueléticos y la productividad. En contraste, no recomiendan combinarlos con la capacitación en manejo del estrés laboral, por no encontrar un efecto significativo^(69)^.

Aunque los resultados son positivos, es importante considerar que la ergonomía se centra en adaptar las condiciones laborales y en identificar riesgos, siendo solo un indicador de un problema mayor en el lugar de trabajo^(20)^.

Otras estrategias, descritas en estudios no incluidos en esta revisión, reportan que ajustar los horarios de trabajo puede reducir el riesgo de sufrir dolor en los hombros. Evitar las jornadas laborales excesivamente prolongadas (más de 46 horas semanales) puede prevenir el dolor en cuello y hombros^(70)^. La disponibilidad y el uso de equipos mecánicos para movilizar y trasladar pacientes se asocian con una reducción en el riesgo de dolor en los hombros^(71)^. Algunas revisiones sistemáticas^(15,72)^ han reportado que el uso de ascensores mecánicos o deslizantes se relaciona con una reducción en la prevalencia de lesiones y dolor relacionados con el trabajo en profesionales de la salud, entre los que se encuentran los de Enfermería. Otros estudios^(2,73-74)^ refieren que factores como turnos discontinuos/nocturnos y trabajar en áreas como quirófanos, servicio de cirugía, urgencias, unidad de cuidados intensivos, ginecología y obstetricia^(75-78)^ se han relacionado con un mayor riesgo de DME. Por ello, los directivos deben de considerar estas variables al planificar la distribución de los servicios y turnos, adaptándolos a las necesidades individuales del personal con DME.

En esta investigación no se recuperaron estudios experimentales que sustenten el efecto de las adecuaciones ergonómicas, así como de implementaciones o cambios en la infraestructura, mejoras de espacios físicos, mobiliario ergonómico o estrategias de dotación de personal y organización de los servicios/procesos en el lugar de trabajo. Estas intervenciones podrían contribuir a reducir los TME y el DME, así como facilitar la rehabilitación o garantizar una reincorporación segura a las actividades laborales^(24,79-80)^. Es posible que la falta de evidencia sólida se deba a los costos asociados y a las políticas de gestión organizacional de los hospitales. No obstante, es relevante considerar estas necesidades para brindar una atención integral en el ambiente laboral del personal de Enfermería y hacer posible la adopción de estrategias de intervención más eficaces.

Se destaca la importancia de implementar intervenciones efectivas en el entorno laboral hospitalario para prevenir que los profesionales de Enfermería desarrollen TME, así como para garantizar que quienes ya presentan DME puedan continuar trabajando en condiciones dignas, en un ambiente adaptado a sus condiciones físicas y psicológicas.

Limitaciones: la cantidad limitada de estudios experimentales y pre-experimentales sobre el tema, la calidad de las metodologías, el pequeño tamaño muestral, la cantidad de estudios con riesgo de sesgo y la cantidad de intervenciones implementadas fueron factores limitantes para un análisis más profundo de los resultados, lo que impidió un metaanálisis. Dada la especificidad de la población de Enfermería en la identificación de los estudios, es posible que se hayan omitido algunos trabajos realizados con otros profesionales de la salud, incluyendo profesionales de Enfermería.

## Conclusión

Esta revisión demostró que las estrategias más efectivas son aquellas que combinan las acciones individuales de los profesionales de Enfermería y mejoras en las condiciones de trabajo considerando aspectos físicos, psicosociales y ergonómicos para adaptar el entorno laboral, prevenir la progresión del dolor musculoesquelético y mantener el desempeño laboral.

Las estrategias más efectivas para afrontar el DME en los profesionales de Enfermería hospitalaria son el ejercicio físico, los estiramientos, la auriculoterapia y el *mindfulness*. Es fundamental promover estas estrategias para el autocuidado, principalmente en entornos hospitalarios con jornadas prolongadas y en servicios que generan más carga física y emocional. Las estrategias más efectivas relacionadas con el ambiente laboral hospitalario son aquellas que adoptan un enfoque integral y que combinan aspectos físicos, psicosociales y ergonómicos, dirigidos tanto a empleados como a empleadores. Esto puede prevenir la progresión del DME y permite que los profesionales puedan continuar desempeñando sus actividades laborales.

A futuro, es crucial llevar a cabo investigaciones del tipo ECA que evalúen de manera integral estrategias encaminadas a las necesidades individuales, terapias alternativas y adaptaciones especificas en los lugares de trabajo para beneficiar a los profesionales de Enfermería que sufren DME.

## Data Availability

Todos los datos generados o analizados durante este estudio están incluidos en este artículo publicado.
